# Joint Efforts of Replicative Helicase and SSB Ensure Inherent Replicative Tolerance of G‐Quadruplex

**DOI:** 10.1002/advs.202307696

**Published:** 2023-12-21

**Authors:** Lijuan Guo, Yanling Bao, Yilin Zhao, Zhiyun Ren, Lulu Bi, Xia Zhang, Cong Liu, Xi‐Miao Hou, Michelle D. Wang, Bo Sun

**Affiliations:** ^1^ School of Life Science and Technology ShanghaiTech University Shanghai 201210 China; ^2^ CAS Center for Excellence in Molecular Cell Science, Shanghai Institute of Biochemistry and Cell Biology Chinese Academy of Sciences Shanghai 200031 China; ^3^ University of Chinese Academy of Sciences Beijing 100049 China; ^4^ Interdisciplinary Research Center on Biology and Chemistry, Shanghai Institute of Organic Chemistry Chinese Academy of Sciences Shanghai 201210 China; ^5^ College of Life Sciences Northwest A&F University Yangling Shaanxi 712100 China; ^6^ Department of Physics, Laboratory of Atomic and Solid State Physics Cornell University Ithaca NY 14853 USA; ^7^ Howard Hughes Medical Institute Cornell University Ithaca NY 14853 USA

**Keywords:** DNA replication, G‐quadruplex, helicase, polymerase, single molecule, SSB

## Abstract

G‐quadruplex (G4) is a four‐stranded noncanonical DNA structure that has long been recognized as a potential hindrance to DNA replication. However, how replisomes effectively deal with G4s to avoid replication failure is still obscure. Here, using single‐molecule and ensemble approaches, the consequence of the collision between bacteriophage T7 replisome and an intramolecular G4 located on either the leading or lagging strand is examined. It is found that the adjacent fork junctions induced by G4 formation incur the binding of T7 DNA polymerase (DNAP). In addition to G4, these inactive DNAPs present insuperable obstacles, impeding the progression of DNA synthesis. Nevertheless, T7 helicase can dismantle them and resolve lagging‐strand G4s, paving the way for the advancement of the replication fork. Moreover, with the assistance of the single‐stranded DNA binding protein (SSB) gp2.5, T7 helicase is also capable of maintaining a leading‐strand G4 structure in an unfolded state, allowing for a fraction of T7 DNAPs to synthesize through without collapse. These findings broaden the functional repertoire of a replicative helicase and underscore the inherent G4 tolerance of a replisome.

## Graphical abstract

G‐quadruplex (G4), a four‐stranded noncanonical DNA structure, has been recognized as a potent block to DNA replication. This study uncovers two G4‐overcoming replication pathways with the involvement of the core replisome only. Concisely, the replicative helicase coordinates with the SSB protein to unfold intramolecular G4 structures, paving the way for the advancement of the replication fork.

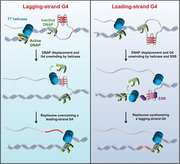

## 1 Introduction

During DNA replication, replisomes inevitably encounter various obstacles, such as DNA damage, DNA‐binding proteins, and DNA secondary structures.^[^ ^1^ ^]^ Failure to effectively address these obstacles results in replication fork arrest and collapse, giving rise to genome instability and chromosomal aberrations.^[^ ^2^ ^]^ To avoid that and ensure timely and accurate DNA replication, cells and viruses have evolved a slew of strategies to overcome these impediments to the replication fork.^[^ ^3^ ^]^ In general, these known pathways can be roughly divided into two categories. In one, a replisome can directly synthesize through interfered DNA by dismantling or enduring the obstacle. These pathways often deploy additional proteins beyond the core replisome, such as helicase, nuclease, and translesion polymerase, to process these obstacles.^[^ ^1^, ^4^ ^]^ Within these pathways, these specialized proteins often remove or accommodate the impediment to the replication fork.^[^ ^5^ ^]^ Alternatively, a replisome can find a way to circumvent an obstacle and continue the replication without processing it.^[^ ^6^ ^]^ For example, in the skipping and re‐priming pathway, a replisome can reinitiate DNA synthesis downstream of an obstacle via a primase‐dependent priming event;^[^ ^7^ ^]^ the fork reversal and template switching pathway permits the utilization of the nascent lagging strand DNA instead of the interfered leading strand as a template for DNA polymerization.^[^ ^6c^ ^]^ How replisomes, once encountering obstacles, select a proper obstacle‐overcoming pathway to continue DNA replication has remained enigmatic. A comprehensive mechanistic view of the response of replisomes to obstacles would enhance our understanding of genomic instability and cancer development.

Non‐B‐form DNA structures are one of the prevalent impediments to the smooth progression of the replisome.^[^ ^1c^ ^]^ G‐quadruplex (G4), one of the stable noncanonical DNA structures, is based on the stacking of two or more G‐quartets, which are circular planar structures formed by four guanines bases connected by Hoogsteen hydrogen bonding.^[^ ^8^ ^]^ According to a genome‐wide sequencing‐based study, at least 716310 potential G4‐forming sequences exist in the human genome.^[^ ^9^ ^]^ Besides positive roles in DNA metabolism,^[^ ^10^ ^]^ there is accumulating in vitro and in vivo evidence that G4 structures threaten progressive DNA replication, causing DNA double‐strand breaks and genome instability.^[^ ^11^ ^]^ Accordingly, a few studies have reported the replicative tolerance of G4.^[^ ^5a^ ^]^ Replisomes can employ G4‐unwinding helicases and SSBs, such as FANCJ and CST, to convert G4 into ssDNA before DNA polymerization.^[^ ^12^ ^]^ Replisomes may also use specialized proteins, such as PrimPol or BRAC1/2, to bypass a G4 without hindering the replication fork.^[^ ^7^, ^13^ ^]^ Despite extensive studies, numerous questions remain to be addressed. For example, what are the inherent abilities of individual replicative proteins and replisome complexes to tolerate G4? Are there alternative G4‐overcoming pathways that do not rely on accessory proteins, and if so, what roles do individual replicative proteins play and how do they coordinate? How do the stability and location of G4 regulate a replisome's tolerance strategies?

In this work, we aimed to address the abovementioned questions using bacteriophage T7 replisome as a model replication system. Within T7 phage, only four proteins are required for primary DNA replication: T7 helicase‐primase (gp4, hereafter referred to as T7 helicase) unwinds DNA and primes DNA synthesis; The gene 5 protein (gp5) complexed with the processivity factor *Escherichia coli* thioredoxin (trx) is responsible for DNA polymerization (hereafter the gp5 and trx complex is referred to as T7 DNAP); The product of gene 2.5 (gp2.5) is a single‐stranded DNA binding protein (SSB) that prevents DNA secondary structure formation.^[^ ^14^ ^]^ We found that T7 DNAP preferentially binds to the fork junction generated by the formation of an intramolecular G4, which impedes the synthesis of T7 DNAP. Nevertheless, T7 helicase can displace fork‐bound inactive DNAPs and resolve lagging‐strand G4s, paving the way for DNA synthesis. Moreover, whereas gp2.5 alone cannot unfold G4, it can assist T7 helicase in preserving the unwound state of leading‐strand G4s, allowing T7 DNAP to overcome them. Overall, these results exhibit the inherent G4 tolerance by the T7 replisome and unveil new G4‐overcoming pathways for a replisome. Both the locating strand and the stability of a G4 are determinants of the pathway choice and replicative proteins required for the tolerance.

## 2 Results

### 2.1 Conflicting Outcomes of T7 DNAP in Response to a Leading‐Strand G4

**Figure 1:**
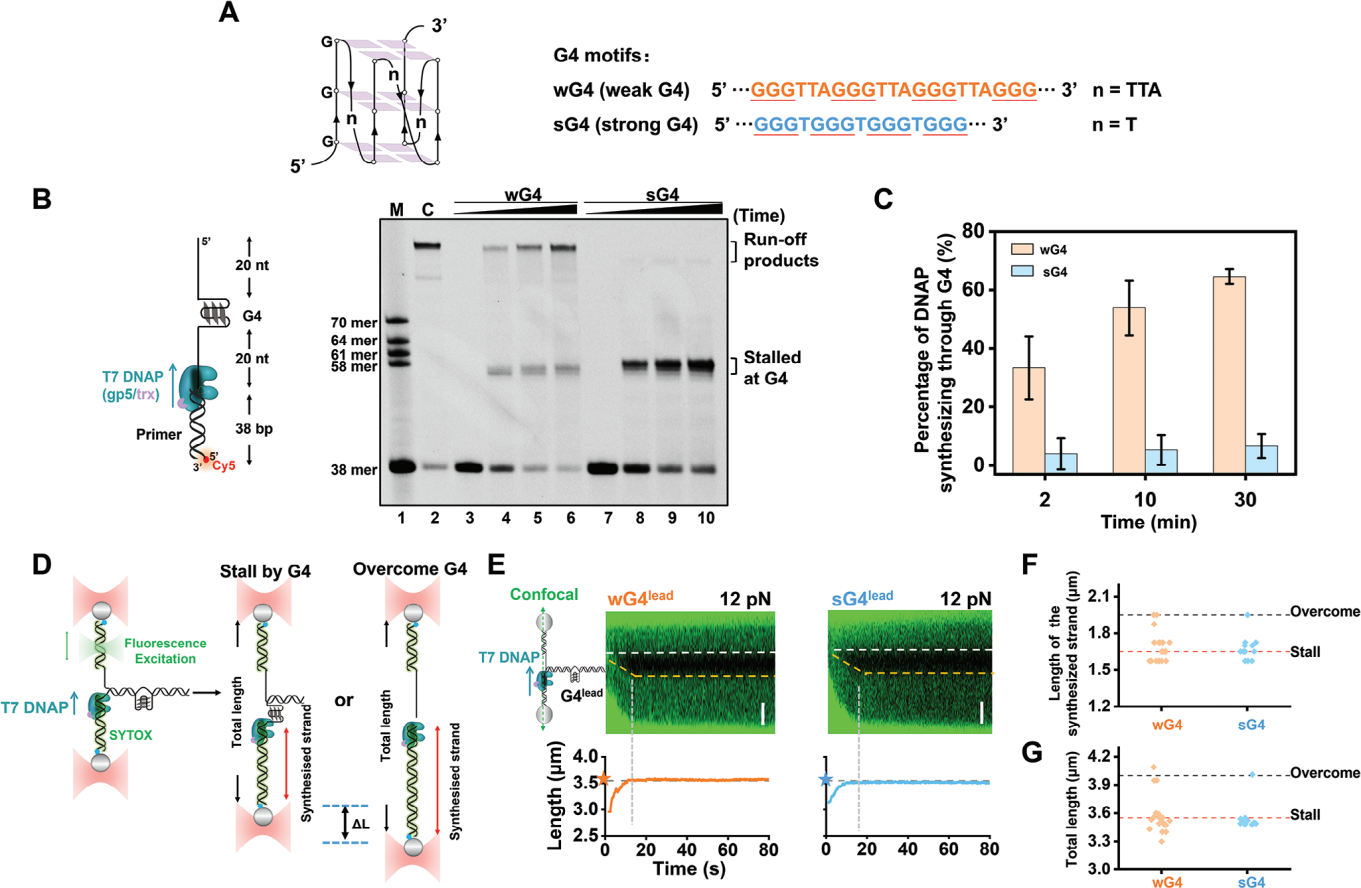
Effect of intramolecular G4 structures on synthesizing T7 DNAP. A) Schematic of a G4 structure and the sequences of the weak G4 (wG4) and strong G4 (sG4) motifs. B) Schematic of the primer extension assay with a G4‐containing DNA template. A 38‐mer primer with Cy5 labeled at the 5′ end is hybridized with the G4 template. Denaturing PAGE analysis of primer extension by T7 DNAP on either a canonical DNA template (no G4, denoted as “C”) or a G4‐containing DNA template is shown. C) The percentage of primers extended over G4. Data are mean ± SD from three independent experiments. D) Schematic diagram of possible outcomes of T7 DNAP synthesis encountering a leading‐strand G4 in the optical tweezer assay. A T‐shaped DNA template harboring a leading‐strand G4 is suspended between two optical traps while a confocal laser is repeatedly scanned along the plane of the DNA template. The DNA extension and fluorescence can reflect DNA synthesis by T7 DNAP. E) Representative kymographs of G4^lead^‐containing DNA templates under 12 pN in the presence of 100 nM T7 DNAP and their corresponding DNA lengths. The pentagram indicates the G4^lead^ position. The white dotted lines indicate the boundary between ssDNA and dsDNA. The yellow dotted lines indicate the position of the replication fork. The scale bar represents 0.5 µm. F) Statistics of the length of the synthesized DNA. G) Statistics of the total DNA length after T7 DNAP synthesizing. The red dotted line indicates the length when T7 DNAP stalls at the G4. The black dotted line indicates the total length once T7 DNAP overcomes G4 and completes the synthesis. n = 21 for wG4 and 12 for sG4.

It has been acknowledged that extraordinary DNA structures on the leading strand are significant obstacles to the progression of the replication fork, as high‐fidelity replicative polymerases alone cannot proceed through them directly.^[^ ^15^ ^]^ Thus, our study aimed to investigate how a synthesizing T7 DNAP responds to a G4 structure and how G4 stability regulates the outcomes. To this end, we prepared two DNA sequences containing G4 motifs with different loop sequences (n) (**Figure** **1**A and Table S1, Supporting Information). Given that G4 stability is inversely correlated with the loop length,^[^ ^16^ ^]^ we referred to the 87 mer (n = T) and 99 mer (n = TTA) oligonucleotides as sG4 (strong G4) and wG4 (weak G4) templates, respectively (Figure 1A). The prefixes “s” and “w” indicate two G4 structures with distinct stabilities. Under the DNA replication condition, we confirmed that the G4 structures were thoroughly formed within these DNA templates (Figure S1, Supporting Information). A 38‐nt Cy5‐labeled primer was utilized for the primer extension assay. Control experiments showed that T7 DNAP could fully extend the primers on a canonical B‐form DNA template (99 mer) in 10 min without obvious pausing (Figure 1B, lane 2). In stark contrast, on the sG4‐containing template, a negligible amount of the primers was fully extended in 30 min, and 95% of them were stalled at positions 57∼58, just before the G4 position (from 59 to 74) (Note that only primers extended to the G4 position and beyond were calculated in statistics) (Figure 1B,C, lanes 8–10). Few stalled bands before G4 are possible because of the exonuclease activity of wild‐type T7 DNAP that provides a kinetic pathway to reverse DNA synthesis.^[^ ^17^ ^]^ On the other hand, on the wG4‐containing template, up to 65% of the primers were fully extended by T7 DNAP within 30 min, although the rest stalled at G4 (Figure 1B,C, lanes 4–6). Therefore, whereas sG4 presents a substantial impediment, T7 DNAP alone can disrupt and synthesize through relatively weak G4 structures in the primer extension assay.

In light of the results from the primer extension assay, it is posited that a G4‐destabilizing force would assist T7 DNAP in overcoming G4. We employed an optical tweezers‐based strand displacement DNA synthesis assay to test this hypothesis.^[^ ^18^ ^]^ In brief, a T‐shaped DNA template harboring a leading‐strand G4 (G4^lead^, hereafter the superscripts “lead” and “lag” indicate the strand that G4 is located on) was suspended between two optical traps while a confocal laser repeatedly scanned along the plane of the DNA template (Figure 1D; Figure S2, Supporting Information). The formation of the G4 structures within dsDNA was guaranteed by the unpaired ssDNA to the G4 motif (Figure S1, Supporting Information).^[^ ^19^ ^]^ Strand displacement DNA synthesis would result in an extension in DNA length under a constant force of 12 pN (Figure S3, Supporting Information). Meanwhile, we used the fluorescent agent SYTOX as a dsDNA probe to simultaneously monitor the newly synthesized dsDNA. The tension on the DNA was supposed to aid in destabilizing the fork junction and the G4 structure, thus promoting the progression of T7 DNAP.^[^ ^20^ ^]^ However, regarding the sG4^lead^‐containing DNA template, both DNA extension and fluorescence signals suggested that almost all examined T7 DNAPs (10 out of 11 traces) were stalled by sG4^lead^, in agreement with the primer extension assay (Figure 1B,E–G). To our surprise, T7 DNAPs were also found to be mostly impeded by wG4^lead^ in this assay (18 out of 21 traces) (Figure 1E‐G and Figure S4A, Supporting Information), even after a longstanding pause (Figure S4B, Supporting Information). Therefore, the destabilizing force seems not to alleviate the impediment of sG4 to T7 DNAP, and T7 DNAP displayed conflicting responses to wG4^lead^ in the primer extension assay and the strand displacement DNA synthesis assay.

### 2.2 Catalytically Inactive T7 DNAP Bound to G4‐Induced Fork Impedes DNA Synthesis

**Figure 2:**
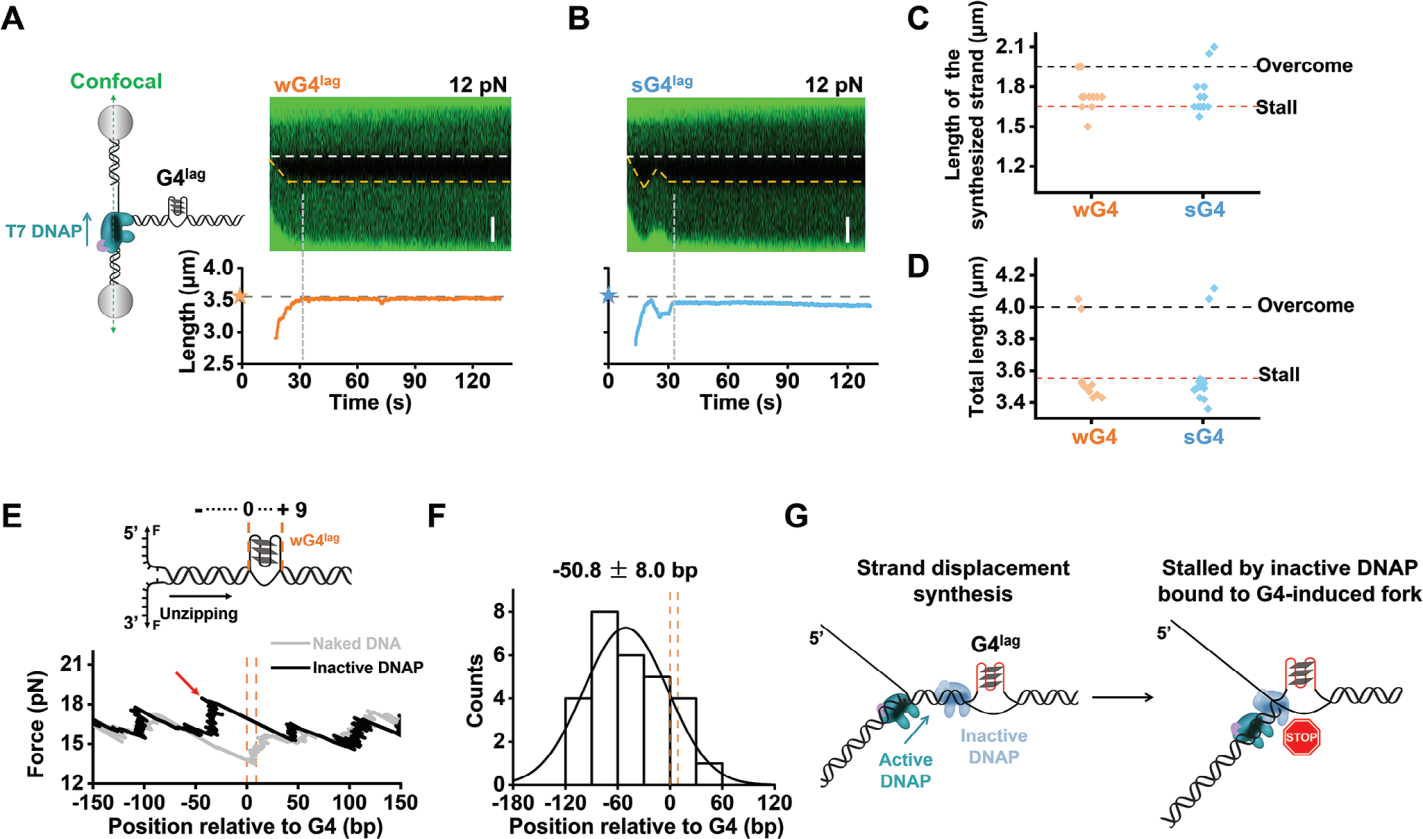
T7 DNAP bound to G4‐induced fork junctions impedes DNA synthesis. A) Schematic of the G4^lag^ template showing the position of the G4 in the DNA (not to scale). A representative kymograph of a wG4^lag^‐containing DNA under 12 pN in the presence of 100 nM T7 DNAP and its corresponding DNA length. The white dotted lines indicate the boundary between ssDNA and dsDNA. The yellow dotted lines indicate the position of the replication fork. The pentagram indicates the wG4 position. The scale bar represents 0.5 µm. B) A representative kymograph of the sG4^lead^‐containing DNA under 12 pN in the presence of 100 nM T7 DNAP and its corresponding DNA length. The scale bar represents 0.5 µm. C) Statistics of the synthesized DNA length. D) Statistics of the total DNA length after T7 DNAP synthesizing. The red dotted line indicates the length when T7 DNAP stalls at the G4. The black dotted line indicates the total length once T7 DNAP overcomes G4 and completes the synthesis. n = 11 for wG4 and 14 for sG4. E) Representative trace of unzipping experiment in the presence of inactive DNAP showing the force versus number of base pairs unzipped. The gray curve corresponds to unzipping naked DNA. The red arrow indicates a force peak above the naked DNA baseline. Red arrows indicate the force peaks. The orange dotted lines indicate the wG4^lag^ location. F) Histogram of the positions of DNAP‐DNA interactions along the DNA sequence. The data and their Gaussian fits are shown. G) Schematic illustration shows that the binding of inactive T7 DNAP at the G4‐induced fork junction blocks the synthesis of an active T7 DNAP.

We tried reconciling the contradictory results regarding T7 DNAP's responses to wG4. Revisiting the primer extension assay with a DNA template containing dsDNA downstream of a wG4 revealed that T7 DNAP can still synthesize through the wG4 (Figure S5, Supporting Information). This finding excludes the possibility that the downstream dsDNA affects T7 DNAP's response to wG4.^[^ ^21^ ^]^ Examining the DNA configurations in the two types of experiments raised another possibility that the G4‐induced fork junctions, composed of the G4 strand, the opposite ssDNA strand and their adjacent intact dsDNA, might be the reason for the impediment detected in the strand displacement DNA synthesis assay. This hypothesis is supported by the finding that T7 DNAP can still overcome G4 in the optical tweezers assay if we disrupt the G4‐induced fork in advance (Figure S6, Supporting Information). To further corroborate that, we designed two DNA templates with a wG4 or sG4 located on the lagging strand, termed wG4^lag^ and sG4^lag^ templates (**Figure** **2**A,B). Intuitively, a leading‐strand T7 DNAP would not be impeded by these lagging‐strand G4s unless the existing G4‐induced forks contribute to the impediment. With these two DNA templates, we found that most T7 DNAPs also stalled at wG4^lag^ (9 out of 11 traces) and sG4^lag^ (12 out of 14 traces) under 12 pN in the strand displacement DNA synthesis assay (Figure 2A–D). We also assayed with a 9‐bp unpaired “bubbled” DNA template containing embedded fork junctions in the replicating DNA but not G4 (Figure S2, Supporting Information). It turned out that T7 DNAP is also incapable of overcoming these DNA “bubbles” (Figure S7, Supporting Information), underlining the importance of the embedded fork in impeding DNA synthesizing by T7 DNAP.

How does the existence of an embedded fork junction influence a synthesizing T7 DNAP? We posited that the formation of the fork junction may incur the binding of T7 DNAP that interferes with the synthesis of DNAPs. To test this hypothesis, we employed an optical tweezer‐based DNA unzipping assay to accurately and precisely determine the position and strength of DNA‐bound proteins.^[^ ^22^ ^]^ For data presentation, we designated the sequence of the displaced ssDNA by G4 formation as 0 to + 9 (Figure 2E). Compared with the unzipping signature of the naked wG4^lag^ DNA template, the addition of T7 DNAP caused 78% of the trajectories to rise in the unzipping force around the G4 position (n = 36) (Figure 2E). The disruption force is located around –50.8 ± 8.0 bp and averaged 18.4 ± 0.6 pN (Figure 2F; Figure S8, Supporting Information). This rise in force was also detectable with the G4^lead^ and the “bubbled” templates, supporting the binding of T7 DNAP to the fork junction (Figure S9, Supporting Information).

Collectively, we provide evidence that the fork junctions arising from the formation of a leading‐ or lagging‐strand G4 incur the binding of inactive DNAPs. In addition to G4, these inactive DNAPs pose intense obstacles to synthesizing DNAPs (Figure 2G).

### 2.3 T7 Helicase Can Resolve G4 Structures and Dismantle Inactive T7 DNAPs

**Figure 3:**
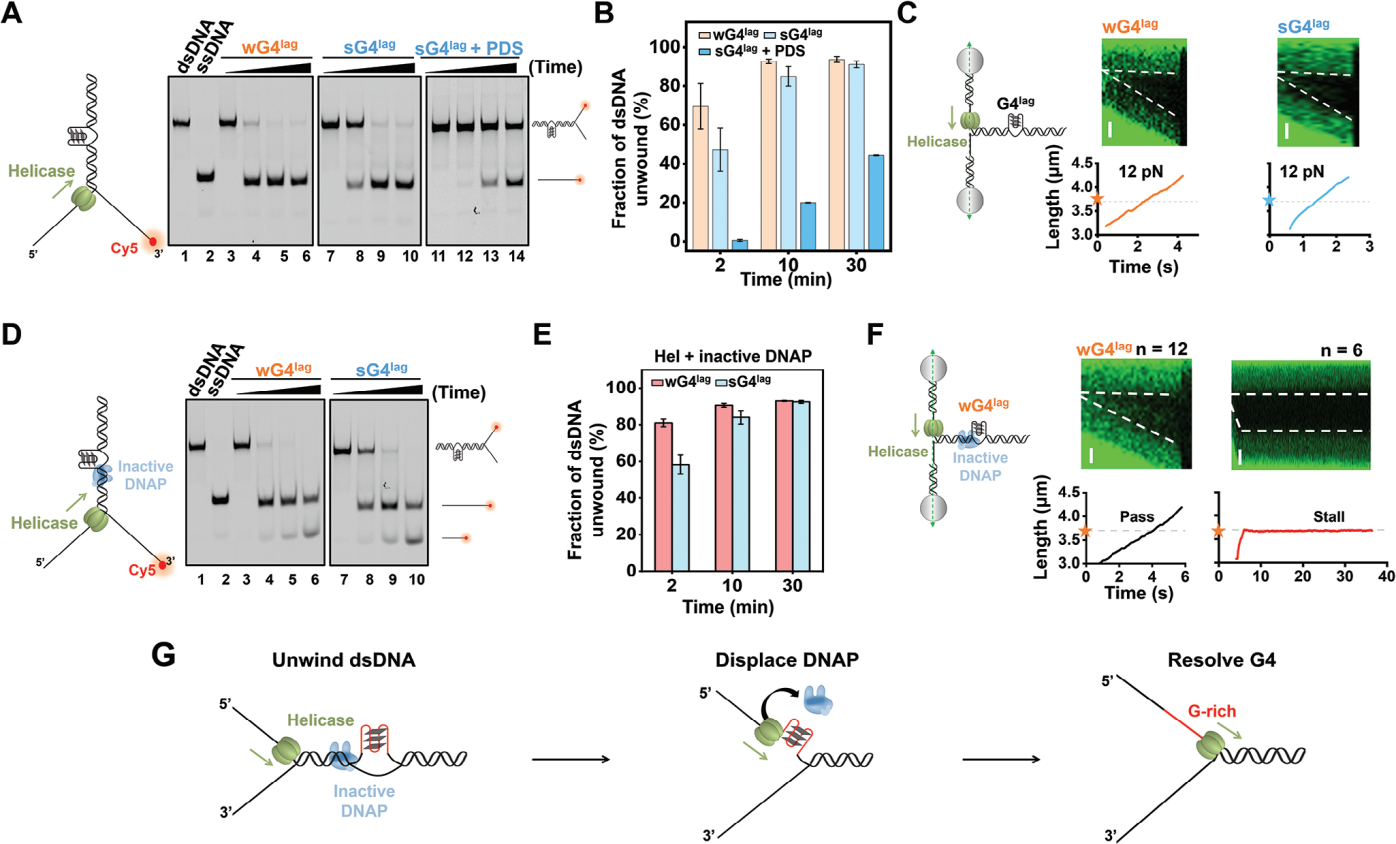
G4 unwinding and DNAP dismantlement by T7 helicase. A) Representative gels of G4‐containing forked DNA unwinding by T7 helicase under indicated incubation time. B) Quantitative analyses of T7 helicase unwinding products. Data are mean ± SD from three independent experiments. C) Schematic of the G4^lag^ template showing the position of the G4 in the DNA (not to scale). Representative kymographs of a G4^lag^‐containing DNA under 12 pN in the presence of 10 nM T7 helicase hexamer and its corresponding DNA length versus time. The orange pentagram indicates the G4 location. The white dotted lines indicate the boundary between ssDNA and dsDNA. The scale bar represents 0.5 µm. D) Representative gel of G4‐containing DNA forked unwinding by T7 helicase in the presence of inactive DNAP under the indicated incubation time. E) The percentage of unwinding products in the presence of inactive DNAP. Data are mean ± SD from three independent experiments. F) Representative kymographs of a wG4^lag^ DNA under 12 pN in the presence of 10 nM hexamer T7 helicase hexamer and 100 nM inactive DNAP and the corresponding DNA length. The scale bar represents 0.5 µm. G) Schematic illustrations show that the T7 helicase dismantles inactive T7 DNAP and subsequently resolves the G4 structure.

The well‐established role of replicative helicases is to catalyze strand separation during DNA replication. Nevertheless, emerging evidence supports their additional functions, such as obstacle dismantlement and DNAP coordination.^[^ ^23^ ^]^ We thus examined the abilities of T7 helicase to resolve G4. Since T7 helicase mainly tracks along the lagging strand of DNA during replication, we first constructed forked DNA templates with a G4 located on the lagging strand (G4^lag^). Meanwhile, the 3′ end of the hybridized ssDNA was labeled with Cy5 for non‐denaturing polyacrylamide gel detection (**Figure** **3**A). We found that T7 helicase can unwind this forked dsDNA template, and the fraction of unwound dsDNA increased over time (Figure 3A, lanes 3–10 and B). Surprisingly, T7 helicase also displayed comparable unwinding activities toward both sG4^lag^‐ and wG4^lag^‐containing dsDNA templates, suggesting G4 resolution by T7 helicase (Figure 3B). One may argue that in addition to resolving the G4 structure, T7 helicase may circumvent it during dsDNA unwinding. Nevertheless, this notion contradicts the finding that the presence of Pyridostatin (PDS), a G4 stabilizer,^[^ ^24^ ^]^ significantly reduced the fraction of the unwound sG4^lag^ dsDNA (Figure 3A, lanes 7–14 and 3B). Therefore, T7 helicase, resembling DNA repair helicases,^[^ ^25^ ^]^ can unwind G4 structures.

We performed the single‐molecule DNA unwinding assay to gain dynamic insight into T7 helicase unwinding through G4.^[^ ^26^ ^]^ In this assay, two strands of a DNA fork junction were held under a constant force that was insufficient to unzip the fork junction mechanically. DNA unwinding by T7 helicase led to the increase in DNA length and the appearance and gradual expansion of the dark region in the kymographs. As expected, the suspended T‐shaped DNA templates were unwound smoothly under 12 pN, and no longstanding stalls or pauses were detected at the G4 position, in agreement with the ensemble assay (Figure 3C). We also carried out a single‐molecule DNA unwinding experiment with the G4^lead^ templates and found that neither sG4^lead^ nor wG4^lead^ are impediments to T7 helicase (Figure S10, Supporting Information).

We further asked whether an unwinding T7 helicase could dismantle inactive T7 DNAPs bound to the G4‐induced fork. In the ensemble DNA unwinding assay, the involvement of T7 DNAP did not alter T7 helicase's unwinding activity on the two G4‐containing DNA templates, and the fraction of unwound dsDNA also increased over time (Figure 3D,E). Although the polymerase activity of T7 DNAP is abolished due to the lack of dNTPs, its exonuclease activity shortened the labeled ssDNA, possibly leading to the inaccurate calculation of the fraction of unwound DNA. We also performed the optical tweezers‐based DNA unwinding assay in the presence of inactive DNAPs. Two‐thirds of examined DNA tethers (n = 18) demonstrated smooth unwinding through G4, though the rest stalled within 1 minute (Figure 3F). Similar results were also obtained with the G4^lead^ DNA templates (Figure S11, Supporting Information). Therefore, although inactive T7 DNAPs bound at the G4‐induced forks add another layer of impediment, T7 helicase still has a chance to dismantle them and subsequently unwind through G4s (Figure 3G).

### 2.4 T7 Helicase Assists T7 DNAP in Synthesizing through G4^lag^ and wG4^lead^

**Figure 4:**
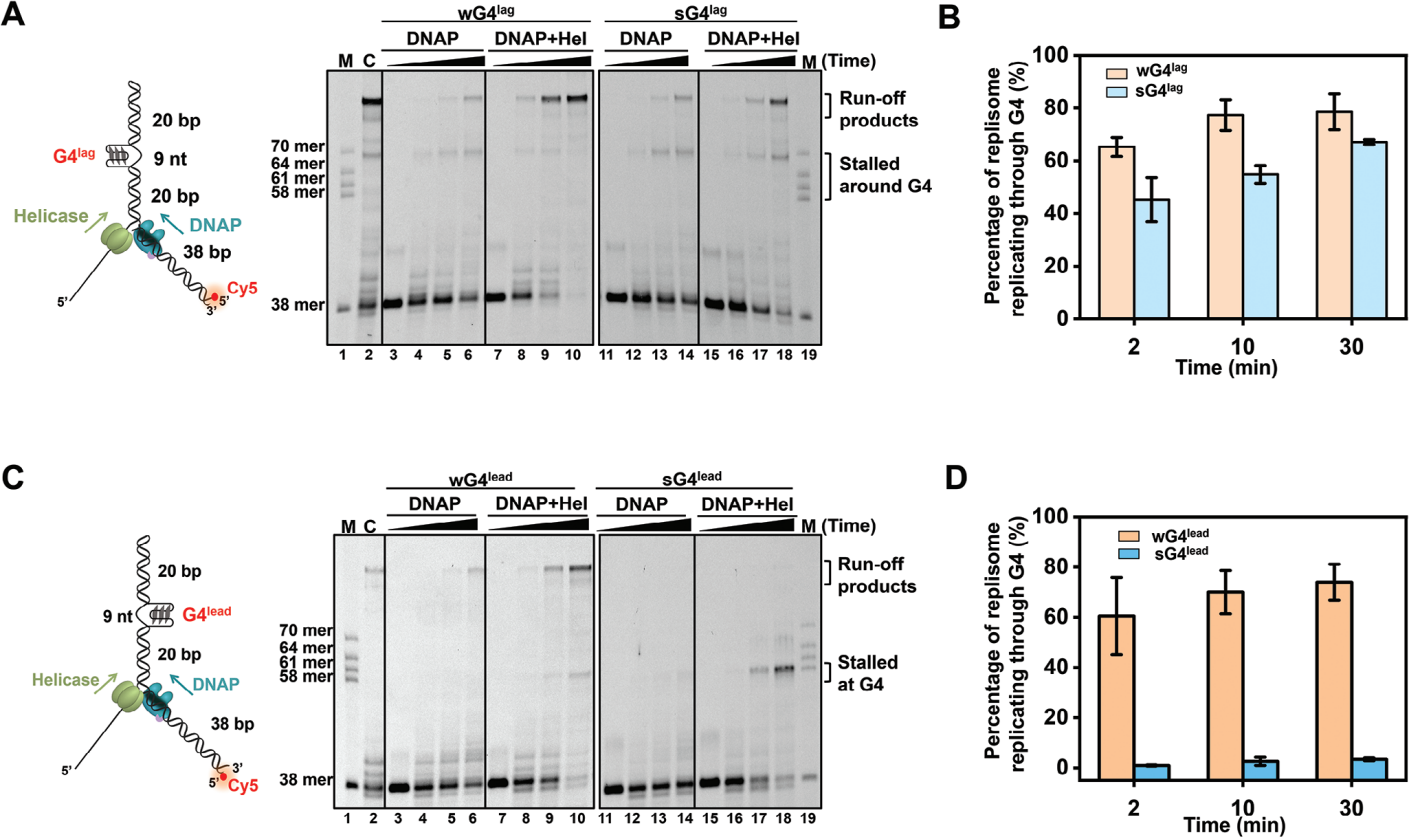
Helicase‐coupled T7 DNAP replicates G4‐containing DNA. A) Schematic illustrating ensemble leading‐strand DNA replication assay on a G4^lag^‐containing template. A denaturing PAGE gel reflects the DNA replication products under the indicated incubation time. B) Quantitative analyses of leading‐strand DNA replication products that overcome the lagging‐strand G4. Data are mean ± SD from three independent experiments. C) Schematic illustrating ensemble leading‐strand DNA replication assay on a G4^lead^‐containing template. A denaturing PAGE gel reflects the DNA replication products under the indicated incubation time. D) Quantitative analyses of leading‐strand DNA replication products that overcome the leading‐strand G4. Data are mean ± SD from three independent experiments.

T7 helicase is known to be tightly coupled with T7 DNAP during DNA replication.^[^ ^23^, ^27^ ^]^ Our previous studies showed that T7 helicase can displace RNA polymerase and assist T7 DNAP in lesion bypass and replication re‐initiation.^[^ ^18^, ^28^ ^]^ Given T7 helicase's abilities to dismantle inactive DNAPs and unwind through G4s, we next asked whether it could assist T7 DNAP in G4 tolerance. To address that, we conducted a series of ensemble leading‐strand DNA replication experiments with near single‐nucleotide resolution. As illustrated in **Figure** **4**A, a replication fork with a Cy5‐labeled DNA primer was utilized, and a G4 structure was embedded in the lagging strand of the DNA template. The replicating DNA products indicated by the extension of the fluorescently labeled primers were examined using a denaturing polyacrylamide gel. Control experiments verified that T7 DNAP coupled with T7 helicase completed the leading‐strand synthesis on a canonical DNA template within 10 min (Figure 4A, lane 2). In comparison, on a wG4^lag^‐containing DNA template, the helicase‐coupled T7 DNAP extended up to 65% of primers to the expected full‐length product within 2 min and the fraction of the full‐length products accumulated over time (Figure 4A, lanes 7–10 and B). The absence of helicase nearly abolished the replication, underlining the necessity of the helicase activity in replicating this template (Figure 4A, lanes 3–6). Consistently, T7 helicase‐coupled DNAP displayed similar DNA replication activity on a sG4^lag^‐containing DNA template, albeit with slightly less G4‐overcoming efficiency (Figure 4A, lanes 11–18 and B).

Next, we placed a G4 structure in the leading strand of the DNA and examined the replicating DNA products in the ensemble DNA replication experiments (Figure 4C). Helicase‐coupled T7 DNAP can overcome wG4^lead^ as well, and the quantitative analysis revealed that 79% of the primers were extended through wG4^lead^ to the full length in 30 min (Figure 4C, lanes 7–10 and D). However, on a sG4^lead^‐containing template, most of the helicase‐coupled T7 DNAP synthesis stalled before the G4 position, and the full‐length DNA products were negligible (Figure 4C, lanes 15–18 and D). These findings can be reasoned by T7 DNAP's inherent capability to synthesize through wG4 but not sG4, as revealed by our primer extension assay (Figure 1B,C).

Collectively, we demonstrated the capability of the helicase‐coupled T7 DNAP to overcome lagging‐strand G4s as well as weak leading‐strand G4s. However, strong G4 structures on the leading strand are insuperable to the helicase‐coupled T7 DNAPs.

### 2.5 Gp2.5 and T7 Helicase Maintain sG4 in an Unfolded State

**Figure 5:**
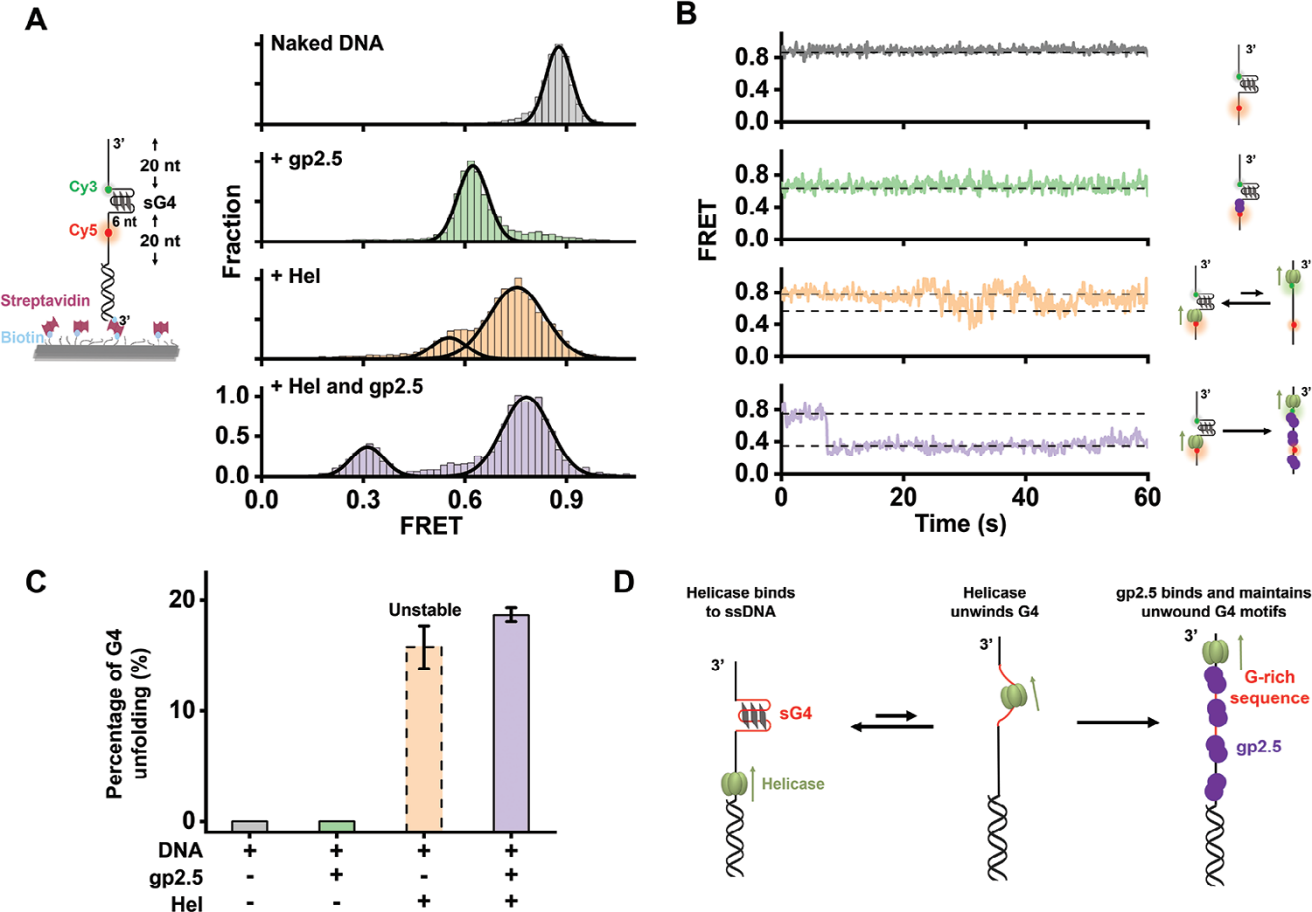
sG4 modulation by T7 helicase and gp2.5. A) Schematic of the smFRET experiment template showing the position of the G4 in the DNA (not to scale). FRET histograms of sG4 substrate in the absence or presence of 2 µM gp2.5 and/or 30 nM T7 helicase hexamer. Gaussian fitting to the histograms is shown. B) Representative single‐molecule trajectories of the sG4 substrate in the absence or presence of 2 µM gp2.5 and/or 30 nM T7 helicase hexamer. C) Fractions of unfolded sG4 in indicated conditions monitored by smFRET. Data are mean ± SD from three independent experiments. For all smFRET experiments, a minimum of 300 smFRET trajectories from three independent experiments are collected for the analysis. D) Schematic illustration of the T7 helicase/gp2.5‐mediated sG4 destabilization.

In addition to T7 helicase and T7 DNAP, gp2.5 also plays essential roles in T7 phage replication.^[^ ^14b^ ^]^ As a ssDNA binding protein, gp2.5 protects ssDNA and coordinates events at the replication fork through physical interactions with T7 DNAP and T7 helicase.^[^ ^14b^ ^]^ SSB proteins, such as RPA and CST, have been reported to regulate G4 structures.^[^ ^29^ ^]^ Thus, we asked whether gp2.5 could modulate G4 structures. We designed a smFRET assay to monitor the potential regulation of sG4 by gp2.5. In this assay, a donor‐acceptor FRET pair was positioned at either side of the sG4 motif (Cy5 is located 6 nt to the 5′ end of the G4 motif, and Cy3 is 1 nt close to its 3′ end) (**Figure** **5**A). This fluorescently labeled ssDNA was hybridized with a 3′ biotin‐labeled oligonucleotide for surface anchoring. Unfolding the compact sG4 structure would increase the end‐to‐end distance of the sG4 motif, therefore decreasing the FRET efficiency, *E*. As expected, the sG4 formation corresponds to a high *E* state of ≈0.87 (Figure 5A,B), and the binding of gp2.5 to the completely unfolded G4 motif gave rise to a low FRET of ≈0.40 (Figure S12B, Supporting Information). However, the introduction of 2 µM gp2.5 into this DNA template containing a preformed sG4 caused a decrease in *E* from ≈0.87 to ≈0.64, which was validated to stem from the binding of gp2.5 to the G4‐adjacent ssDNA instead of sG4 unfolding (Figure S13, Supporting Information). Therefore, gp2.5 alone is incapable of unfolding sG4.

T7 helicase has been demonstrated to resolve strong G4 structures in the ensemble assay (Figure 3A–C). We thus examined the dynamic unfolding of sG4 by T7 helicase in the smFRET experiments. In the presence of T7 helicase, the DNA template exhibited two *E* states with a predominant FRET population at ≈0.75 and a relatively minor population at ≈0.56 (Figure 5A). Based on the results from the control experiments (Figures S12A and S14, Supporting Information), these two states are attributable to the binding of T7 helicase to the G4‐adjacent ssDNA (*E*≈0.75) and the complete G4 unfolding (*E*≈0.56), respectively. The appearance of the FRET state of 0.56 reinforces the conclusion that T7 helicase can unwind sG4. Consistently, single‐molecule trajectories showed frequent transitions between the two *E* states (Figure 5B,C). These transitions possibly arise from the transient G4 unfolding and subsequent refolding, indicating unstable unwinding events.

We further explored the modulation of sG4 by both T7 helicase and gp2.5. When T7 helicase and gp2.5 were sequentially introduced to the DNA template, two dominated *E* states centered at 0.78 and 0.33 were detected (Figure 5A). The low *E* state is comparable to the two proteins binding to the unwound G4 motif (Figure S12C, Supporting Information), indicating that a fraction of the G4 structures was unfolded in the presence of T7 helicase and gp2.5. Consistently, single‐molecule FRET trajectories displayed a quick decrease in *E* from 0.78 to 0.33 and the lower *E* state was retained (Figure 5B,C). These findings favor a model where T7 helicase transiently unwinds sG4, followed by the gp2.5 binding to the unfolded G4 motif, maintaining it in an unfolded state (Figure 5D). The relatively low fraction of unfolded G4 motifs can be explained by the pre‐binding of gp2.5 to the DNA template that prevents the G4 unwinding by T7 helicase (Figure S15, Supporting Information).

### 2.6 T7 Helicase and SSB Aid T7 DNAP in Synthesizing through sG4^lead^

**Figure 6:**
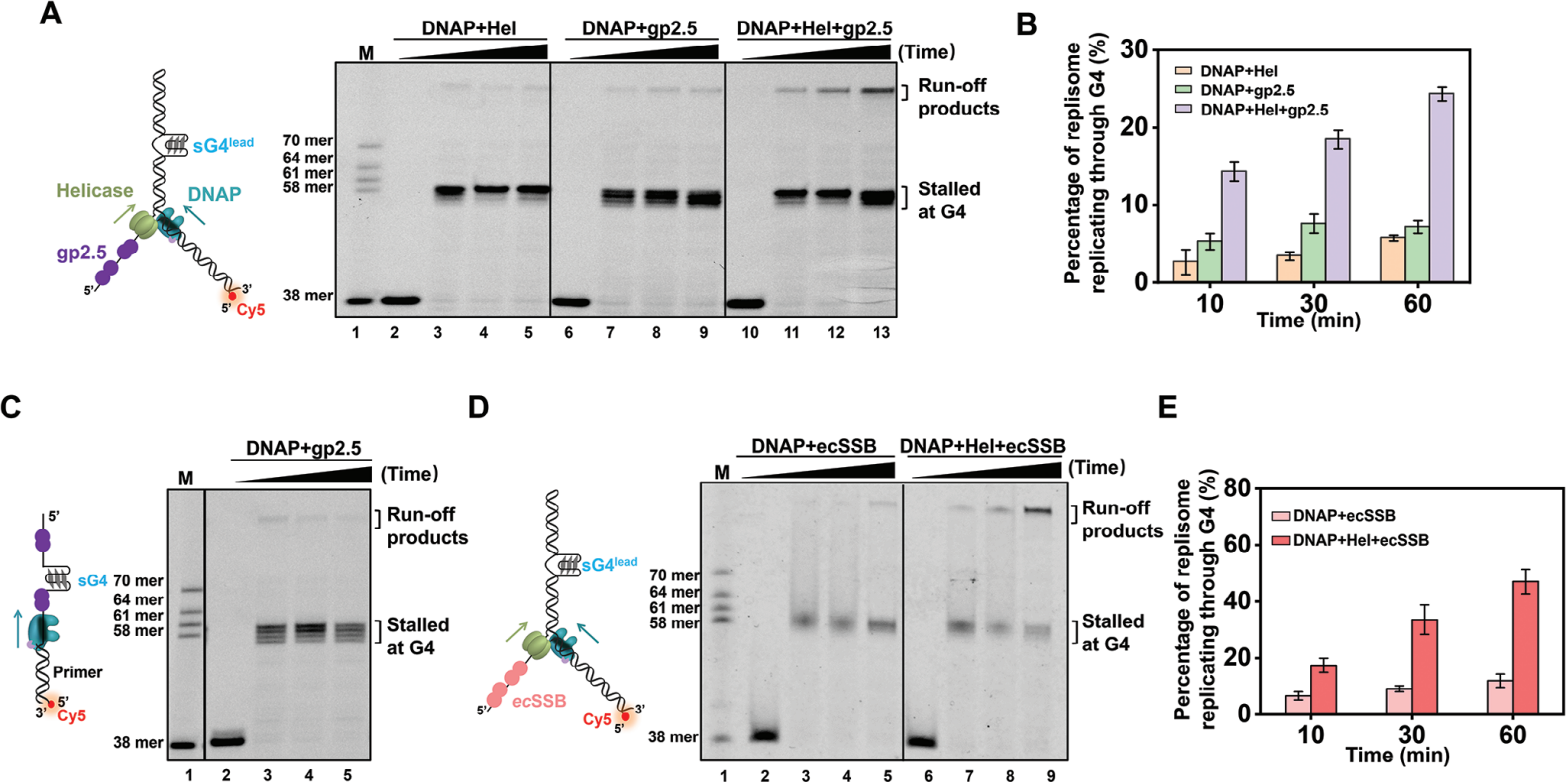
T7 DNAP replicates sG4^lead^‐containing DNA in the presence of T7 helicase and SSB proteins. A) Schematic illustrating the ensemble leading‐strand DNA replication assay with a sG4^lead^‐containing template in the presence of T7 helicase, T7 helicase and/or gp2.5 and a representative gel under indicated conditions is shown. B) The percentage of G4‐overcoming replication products in 10, 30, and 60 min. Data are mean ± SD from three independent experiments. C) Schematic of the primer extension on a sG4‐containing DNA template. Denaturing PAGE of the primer extension by DNAP in the presence of gp2.5 is shown. D) Schematic illustrating the ensemble leading‐strand DNA replication assay with a sG4^lead^‐containing template in the presence of T7 helicase, T7 helicase and/or *E.coli* SSB (ecSSB) and a representative gel under indicated conditions is shown. E) The percentage of G4‐overcoming replication products in 10, 30, and 60 min. Data are mean ± SD from three independent experiments.

The cooperation between T7 helicase and gp2.5 ensures the stable unfolding of a fraction of sG4 structures. This finding motivated us to examine whether the two proteins could help T7 DNAP overcome sG4^lead^. Through the ensemble leading‐strand DNA replication assay, we found that the presence of gp2.5 or T7 helicase barely helped T7 DNAP in counteracting the sG4^lead^ barrier since the majority of the leading‐strand primers were extended to the positions before sG4^lead^ (**Figure** **6**A,B). However, surprisingly, T7 helicase and gp2.5 significantly promoted the sG4^lead^ tolerance by T7 DNAP, and 24% of T7 DNAPs were detected to overcome sG4 within 1 h (Figure 6A,B). Therefore, with the assistance of T7 helicase and gp2.5, T7 DNAP can tolerate sG4^lead^ and directly synthesize through it. We performed the primer extension assay to examine the specific contribution of gp2.5 and concluded that gp2.5 itself cannot stimulate T7 DNAP in G4 overcoming (Figure 6C),^[^ ^30^ ^]^ underscoring the importance of the joint contributions of both T7 helicase and gp2.5 to the G4 tolerance by T7 DNAP. We also carried out the ensemble leading‐strand DNA replication assay with the wG4^lead^‐containing DNA. However, no apparent enhancement in wG4^lead^‐overcoming efficiency of T7 DNAP was detected when both T7 helicase and gp2.5 were present (Figure S16, Supporting Information). This difference might be rationalized by the maximal wG4^lead^‐overcoming efficiency (over 75%) realized by the presence of either protein.

Besides gp2.5, the T7 replisome also uses the host *E. coli* SSB protein (ecSSB) to fulfill DNA replication.^[^ ^31^ ^]^ T7 gp2.5 and ecSSB contain functional homology with an acidic carboxyl terminus that facilitates interactions with other DNA replication proteins, and both can stimulate T7 DNAP activity and allow T7 helicase to load onto ssDNA.^[^ ^32^ ^]^ We replaced the gp2.5 with ecSSB in the leading‐strand DNA synthesis assay and found that ecSSB and T7 helicase can also simulate T7 DNAP synthesizing through sG4^lead^ (Figure 6D,E).

## 3 Discussion

This work delved into how individual replicative proteins within the T7 replisome respond to a preformed intramolecular G4. Unlike eukaryotic replicative polymerases, e.g. pol α、pol δ and pol ε, which struggle to overcome G4 on their own,^[^ ^33^ ^]^ T7 DNAP itself has the intrinsic ability to disrupt and synthesize through weak G4 structures when the complementary DNA strand is absent (Figure 1). However, an intramolecular G4 structure within dsDNA necessarily results in the formation of a “bubbled” DNA structure, within which two fork junctions at both “bubble” ends are formed. The G4‐induced fork junctions mimic a ss‐dsDNA junction and invite the binding of T7 DNAP. These inactive T7 DNAPs act as an additional impediment to synthesizing T7 DNAP. A few non‐replicative polymerases, such as Rev1 and PrimPol, were also reported to bind G4, and they facilitate G4 tolerance by either disrupting G4 structures or re‐priming leading‐strand DNA synthesis downstream of the G4.^[^ ^7^, ^34^ ^]^ However, our findings argue that instead of G4, the G4‐induced fork junction incurs the binding of replicative DNAPs (Figure 2). Moreover, in contrast to the assistant role of non‐replicative DNAPs in G4 overcoming, these inactive DNAPs strengthen G4 as an impediment, hindering the efficient progression of DNA synthesis. The collateral alternation of DNA structures due to G4 formation complicates the consequence of a collision between a replisome and a G4.

The resolution of G4 structures has been restricted to a few non‐replicative helicases, such as BLM, FANCJ, and Pif1, and replicative helicases, such as CMG, are commonly reported to be stalled by G4.^[^ ^35^ ^]^ Our previous works demonstrate the multi‐functional roles of T7 helicase in replication, such as the assembly of replication machinery, lesion bypass, and protein displacement.^[^ ^18^, ^28^ ^]^ This work enriches T7 replicative helicase's function in the G4 resolution (Figure 3). To our knowledge, this is the first observation of a replicative helicase unwinding through an intramolecular G4.^[^ ^25^ ^]^ T7 helicase may dismantle inactive DNAPs through two mechanisms. During unwinding, T7 helicase encircles and translocates along a ssDNA strand and displaces the other once it encounters a fork junction.^[^ ^28^ ^]^ Therefore, T7 helicase may actively dislodge inactive DNAPs from the fork. Alternatively, T7 helicase may deploy DNAPs by transiently removing them from the fork while maintaining the association, preparing DNAPs for the subsequent DNA synthesis if required. Although T7 helicase‐mediated G4 resolution is unstable and the G4 motif can re‐form, the transient unwinding of lagging‐strand G4 structures is sufficient to help advance the DNA replication fork. Moreover, the unwound state of a leading‐strand G4 can be stabilized by the presence of gp2.5 (Figure 5). Previous studies showed that gp2.5 functions as a helicase‐loading factor and modestly stimulates the dTTPase and helicase activity of T7 helicase.^[^ ^14b^ ^]^ T7 helicase may benefit from these stimulations to enhance G4 resolution and protein displacement activities.

**Figure 7:**
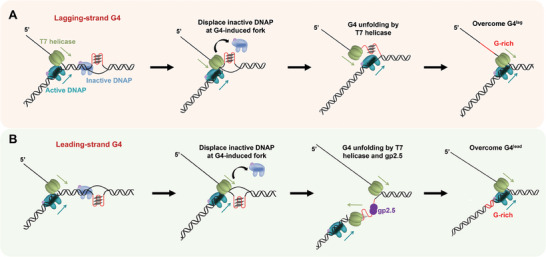
A model of T7 replisome overcoming intramolecular G4s. A) Regarding a G4^lag^‐containing DNA template, the fork junction induced by G4 formation incurs the binding of T7 DNAP and hinders the synthesis of the active DNAP. The T7 helicase can displace the inactive DNAP near the G4 position and resolve the lagging‐strand G4 structure, which assists T7 DNAP in overcoming the obstacle. B) Regarding a G4^lead^‐containing DNA template, T7 helicase dismantles inactive DNAPs and resolves the leading‐strand G4 structure. The gp2.5 binding maintains the leading‐strand G4 motif in an unwound status. The resolution of the leading‐strand G4 structure by T7 helicase and gp2.5 allows T7 DNAP to synthesize through the G‐rich motif.

Our work also provides insights into the mechanism of T7 replisome as a coordinated protein complex in tolerating G4. We proposed two new G4‐overcoming pathways for a replisome based on our findings. The displacement of inactive DNAPs and the resolution of lagging‐strand G4 structures by T7 helicases paved the way for the advancement of the replication fork (**Figure** **7**A). In terms of wG4^lead^, after removing inactive DNAPs by T7 helicase, the leading‐strand T7 DNAP alone can synthesize through a wG4^lead^ structure, ensuring the progression of the replication fork (Figure 4). On the other hand, the resolution of a sG4^lead^ necessitates both gp2.5 and T7 helicase (Figure 5). Unfolding the stable G4 structure requires a strict temporal control of both proteins. The transient G4 unwinding by T7 helicase allows for gp2.5 coating, and the preservation of the unwound state of the G4 motif permits T7 DNAP synthesizing (Figure 7B). In this pathway, the T7 helicase that tracks along the lagging strand may switch to the leading strand for the G4 resolution.^[^ ^36^ ^]^ Notably, no specialized DNA helicases or polymerases are required in both pathways, suggesting that the T7 replisome is inherently permissive to G4 replication. These uncovered pathways also emphasize the importance of the G4 location and stability in regulating the G4‐overcoming pathway choice.

## 4 Experimental Section

### Protein Expression and Purification

T7 DNAP was expressed and purified as previously described.^[^ ^37^ ^]^ The T7 gp5 gene was amplified and constructed in the expression vector pET28a consisting of an N‐terminal His_6_‐tag. The protein was expressed in *E. coli* strain BL21 PlysS (TransGen) cells. The cells were grown to an OD600 of 0.6 before adding 0.4 mM isopropyl‐beta‐D‐thiogalactopyranoside (IPTG). Protein expression was performed at 18 °C for 16 h. Cultures were lysed, and insoluble debris was removed by centrifugation. The Ni‐NTA column (TransGen) further purified the cleared cell lysate from the supernatant. The column was further washed by increasing the imidazole concentration starting from 5 to 20, 100, and finally 300 mM imidazole in elution buffer containing 50 mM Tris‐HCl (pH 7.5), 300 mM NaCl, 0.1 mM EDTA, and 0.1 mM DTT. The majority of T7 gp5 was in the 300 mM imidazole eluate. The following procedure routinely reconstituted T7 DNAP: fresh DTT (0.5 M) was added to a solution of thioredoxin (Sigma‐Aldrich) to a final DTT concentration of 5 mM; The gp5 protein was then combined with reduced thioredoxin in a 1:5 molar ratio and incubated at 4 °C for 1 h. T7 DNAP was stored at −80 °C in the 50 mM Tris‐HCl pH 7.5 buffer, 300 mM NaCl, 0.1 mM EDTA, 3 mM DTT, and 10% (v/v) glycerol.

T7 helicase was expressed and purified as previously described.^[^ ^38^ ^]^ The T7 gp4A^'^ gene was amplified and constructed in an expression vector pET28a consisting of an N‐terminal His_6_‐tag. The purification process for T7 helicase was similar to that of T7 gp5 but included an additional step of gel filtration chromatography using a Superdex 200 Increase 10/30 column (GE Healthcare). T7 helicase was stored at −80 °C in the buffer containing 50 mM Tris‐HCl pH 7.5, 200 mM NaCl, and 10% (v/v) glycerol.

T7 gp2.5 was expressed and purified as follows.^[^ ^39^ ^]^ Briefly, *E. coli* BL21 PlysS cells transformed with pET28a‐gp2.5 were grown to an OD600 of 1 before adding 1 mM IPTG. Cultures were lysed, and insoluble debris was removed by centrifugation. The Ni‐NTA column further purified the cleared cell lysate from the supernatant. The bulk of T7 gp2.5 was in the 300 mM imidazole eluate. T7 gp2.5 was stored at −80 °C in the buffer containing 50 mM Tris‐HCl pH 7.5, 300 mM NaCl, and 10% (v/v) glycerol.

*E. coli* SSB was expressed and purified as follows.^[^ ^40^ ^]^ Briefly, *E. coli* BL21 cells transformed with pET28a‐ecSSB were grown to an OD600 of 0.8 before adding 1 mM IPTG. Protein expression was performed at 37 °C for 3 h. The cleared cell lysate was purified from the supernatant using a Ni‐NTA column by increasing the imidazole concentration in elution buffer containing 20 mM Tris‐HCl pH 7.9, 500 mM NaCl, and 0.1% Triton X‐100. *E.coli* SSB was stored at −80 °C in the buffer containing 20 mM Tris‐HCl pH 8.0, 500 mM NaCl, 1 mM EDTA, and 1 mM DTT).

### Preparation of DNA Templates

For the primer extension assay, a 38‐nt primer and a G4‐containing oligonucleotide were annealed at a 1:1.5 ratio by incubating at 95 °C for 5 min, and then slowly cooling down to room temperature within 3 h in G4 annealing buffer containing 20 mM Tris‐HCl pH 8.0, and 100 mM KCl. For the ensemble leading‐strand DNA replication assay, the DNA template was prepared by annealing a 38‐nt primer, a leading strand DNA, and a lagging strand DNA together at a 1:1.3:4 ratio in the G4 annealing buffer. For the helicase unwinding assay, the DNA template was prepared by annealing a Cy5‐labeled oligonucleotide and a G4‐containing oligonucleotide at a 1:1.5 ratio in the G4 annealing buffer. For the smFRET assay, a biotin‐labeled oligonucleotide and a G4‐containing oligonucleotide were slowly annealed at a 1:1.5 ratio in the G4 annealing buffer.

The T‐shaped DNA template used for the optical tweezers (LUMICKS, Netherlands) was prepared as previously described (Figure S2 and Table S1, Supporting Information).^[^ ^41^ ^]^ The DNA construct comprises three DNA segments – two arms and a trunk‐ linked through two short adapters. The trunk was a ligation product of three DNA segments (upstream segment, G4/9‐bp bubble segment, and downstream segment). The sequences of primers and oligonucleotides used in this work were listed in Table S1 (Supporting Information).

### Ensemble DNA Unwinding and Replication Assays

For the DNA replication assay, T7 DNAP (100 nM) was preassembled on the DNA (3 nM) with or without T7 helicase (30 nM hexamer) in the replication buffer containing 50 mM Tris‐HCl pH 7.5, 50 mM NaCl, 2 mM dTTP, 10% glycerol, and 100 µM dNTPs (each). Reactions were initiated by adding 5 mM MgCl_2_ and were terminated with a quenching buffer containing formamide and 10 mM EDTA, followed by heating to 95 °C for 10 min. The replication products were separated by 12% denaturing polyacrylamide gel electrophoresis (7 M urea PAGE) in TBE and visualized using a Typhoon FLA 9500 (GE Healthcare) with a 635‐nm laser.

For the DNA unwinding assay, T7 helicase (30 nM hexamer) was incubated with DNA (3 nM) after assembling in reaction buffer (2 mM dTTP, 50 mM Tris‐HCl pH 7.5, 50 mM NaCl, 10% glycerol, 2 mM DTT). Unwinding was initiated by adding 5 mM MgCl_2_ and quenched at 0, 2, 10, and 30 min by adding 0.24% SDS, 40 mM EDTA, and 20% glycerol. The products were immediately separated on an 8% native PAGE gel. The gels were scanned using Typhoon FLA 9500, monitoring the Cy5 fluorescence of the labeled primer.

The gels were quantified using ImageJ. For the DNA replication assay, the fraction of DNA replicating through G4 was quantified by the ratio of the full‐length products to the combination of the full‐length products d and the stalled products. For the DNA unwinding assay, the fraction of dsDNA unwinding was similarly quantified. Each experiment was independently carried out at least three times.

### Single‐Molecule Helicase Unwinding and DNAP Strand Displacement DNA Synthesis Assays

A dual‐optical tweezer setup combined with confocal microscopy and microfluidics (LUMICKS, C‐trap) was employed to perform the helicase unwinding and the strand displacement synthesis experiments.^[^ ^18^, ^26^, ^42^ ^]^ A single DNA molecule was captured between two streptavidin‐coated polystyrene beads (1.76 µm in diameter) and was tensioned by increasing the distance between the optical traps. The DNA tether was transported to the protein channel as described for each assay. Unless stated otherwise, all experiments were carried out in the replication buffer with 200 nM SYTOX. A 532‐nm laser was used for the SYTOX imaging.

Helicase unwinding and DNAP strand displacement synthesis experiments were conducted as follows. First, several hundred base pairs of dsDNA were mechanically unzipped (with an average unzipping force of ≈15 pN) at a constant velocity of 0.1 µm s^−1^ to produce a ssDNA loading region for helicase or a priming template for DNAP. Second, DNA length was maintained until a force dropped below a threshold, indicating helicase or polymerase unwinding of the DNA fork. Finally, a constant force was maintained while helicase or polymerase unwound the dsDNA. Kymographs were generated via a confocal line scan through the center of the two beads with a pixel size of 75 nm for 0.2 ms. Force and extension data were taken at 100 Hz. The kymographs from experiments with a shortened DNA template determined the synthesized DNA lengths with DNAP stalling at G4 (Figure S3A, Supporting Information). The total DNA length with T7 DNAP synthesizing through G4 to the end was determined by the experiment wherein T7 DNAP completed the synthesis on a canonical DNA template without G4 (Figure S3B, Supporting Information).

### Single‐Molecule DNA Unzipping Assays

DNA unzipping experiments were performed on optical tweezers. The sample chamber preparation was described previously.^[^ ^41^ ^]^ Biotin‐tagged DNA was added and incubated to form the DNA tethers. Antidigoxin‐coated 0.5‐µm polystyrene microspheres (Polysciences) were then added to the chamber. Finally, the protein solution flowed into the sample chamber before data acquisition. T7 DNAP (200 pM) was inactive due to the absence of dNTPs.

Single‐molecule DNA unzipping experiments data were acquired at 5 kHz and later filtered to 50 Hz. As previously described, the acquired data signals were converted into force and DNA extension.^[^ ^22^ ^]^ One base pair unwound generated two nucleotides of ssDNA for the DNA unzipping studies. Accordingly, real‐time DNA extension was converted into the number of base pairs unwound. The data were then aligned to a theoretical unzipping curve for the mechanically unzipped section of the DNA.

### Single‐Molecule Fluorescence Resonance Energy Transfer (smFRET) Assay

The smFRET assays were performed as previously described.^[^ ^43^ ^]^ For the G4 formation and unwinding assays, a fluorescently labeled DNA substrate (< 50 pM) was immobilized on the glass surface via a streptavidin‐biotin interaction. gp2.5 (2 µM) or T7 helicase (30 nM hexamer) was introduced to the fluorescently labeled G4‐containing DNA. All imaging was performed using total internal reflection fluorescence (TIRF) microscopy in the imaging buffer containing 50 mM Tris‐HCl (pH 7.5), 50 mM NaCl, 10% glycerol, 2 mM DTT, 5 mM MgCl_2_, 1 mg mL^−1^ glucose oxidase, 0.8% D‐glucose, 0.4 mg mL^−1^ catalase, and 4 mM Trolox at room temperature. An EMCCD camera (Andor) was used to record videos at an exposure time of 100 ms for 600 frames. Each frame was further processed to extract single‐molecule fluorescence intensities.

The FRET efficiency of a single molecule was approximated as *E* = *I_A_*/(*I_D_* + *I_A_*), where I_D_ and I_A_ were the background and leakage‐corrected emission intensities of the donor and acceptor, respectively. For histogram analysis, short of (2 s) movies were taken shorts from more than 10 different imaging surfaces, with each movie yielding approximately 200 FRET values after manually filtering photobleaching effects. Data from over 2000 molecules fitted to Gaussian distributions using Origin 2023 were collected to generate smFRET histograms.

### Statistical Analysis

Statistical analysis was performed using the Origin2023 software, and the corresponding statistical information was provided in the experimental section and figure legends. The gels were quantified using ImageJ. All experiments were independently repeated at least three times. Data were presented as mean ± SD.

## Conflict of interest

The authors declare no conflict of interest.

## Author Contributions

L.G. and Y.B. contributed equally to this work. B.S. conceived the project and supervised all research. L.G. and Y.B. conducted all the experiments. L.G., Y.B., M.D.W., and B.S. analyzed and interpreted the data. Y.Z., Z.R., C.L., and X‐M.H. helped with the protein purification. L.B. and X.Z. participated in the DNA template preparation. B.S. and L.G. wrote the manuscript with inputs from all authors.

## Supporting information

Supporting Information

## Data Availability

The data that support the findings of this study are available from the corresponding author upon reasonable request.
