# Supplementary material for: Joint Efforts of Replicative Helicase and SSB Ensure Inherent Replicative Tolerance of G‐Quadruplex
Source: Adv Sci (Weinh). 2023 Dec 21;11(9):2307696. doi: 10.1002/advs.202307696 (PMC10916570; doi:10.1002/advs.202307696)
Supplement: Supplementary file 1 — Supporting Information [file ADVS-11-2307696-s001.pdf]

ADVANCED SCIENCE
Open Access

Supporting Information

for *Adv. Sci.*, DOI 10.1002/advs.202307696

Joint Efforts of Replicative Helicase and SSB Ensure Inherent Replicative Tolerance of G-Quadruplex

*Lijuan Guo, Yanling Bao, Yilin Zhao, Zhiyun Ren, Lulu Bi, Xia Zhang, Cong Liu, Xi-Miao Hou, Michelle D. Wang and Bo Sun**

Supporting Information

for *Adv. Sci.,* DOI 10.1002/advs. 202307696

Joint Efforts of Replicative Helicase and SSB Ensure Inherent Replicative Tolerance of G-Quadruplex

*Lijuan Guo, Yanling Bao, Yilin Zhao, Zhiyun Ren, Lulu Bi, Xia Zhang, Cong Liu, Xi-Miao Hou, Michelle D. Wang, and Bo Sun**

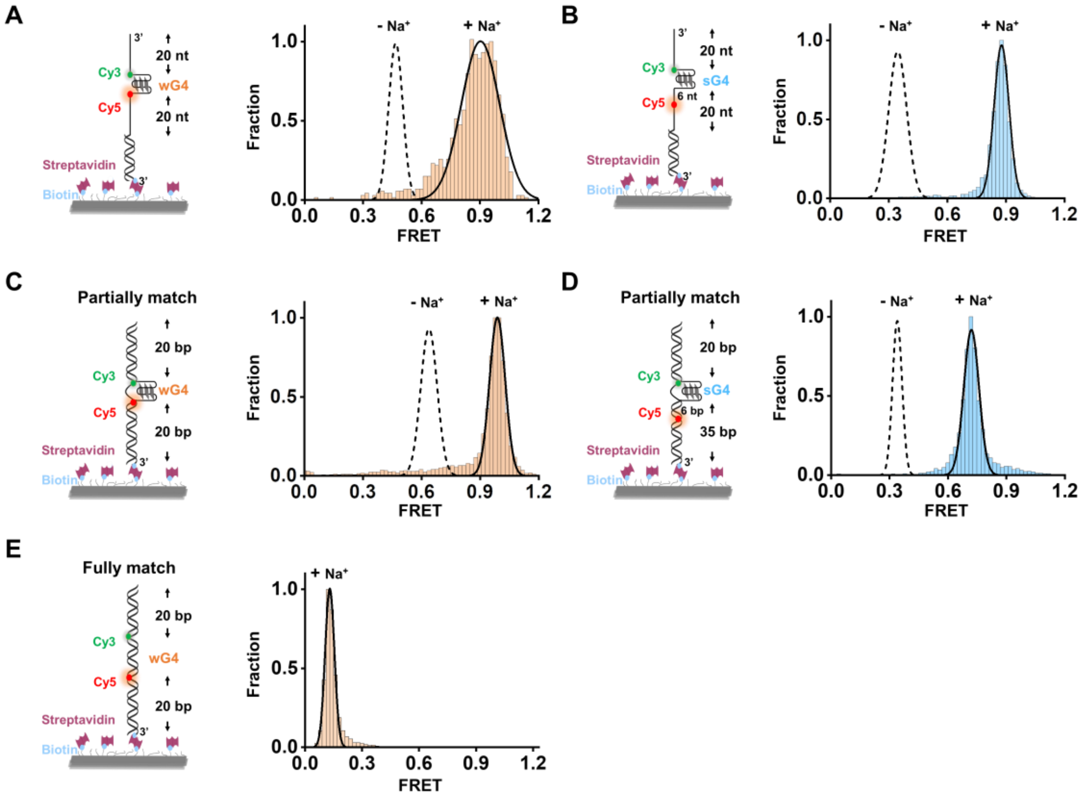

Figure S1. smFRET measurements confirm the formation of G4 structures.

A and B. FRET distributions of the fluorescently labeled ssDNA containing the G4 motif. The distributions show a high *E* value in the replication reaction buffer containing Na^+ and a low *E* value in the Na^+-depleted buffer (dashed Gaussian). **C and D.** FRET distributions of the G4 motif-containing ssDNA hybridized with a partially matched ssDNA. The distributions also show a high *E* value in the reaction buffer containing Na^+ and a low *E* value in the Na^+-depleted buffer (dashed Gaussian). **E.** The FRET distributions of the wG4 motif-containing ssDNA hybridized with a fully matched ssDNA showing a low *E* value in the replication reaction buffer containing Na^+. The DNA sequences used for constructing these DNA templates are listed in **Table S1**.

These findings suggest that in the presence of Na^+, the G4 structure is thoroughly formed before and after the hybridization with a partially matched

ssDNA, as the compact structure shortens the distance between the two fluorophores, giving rise to a high *E* value. However, hybridizing the G4 motif containing DNA with a fully matched ssDNA results in a dsDNA form instead of an embedded G4.

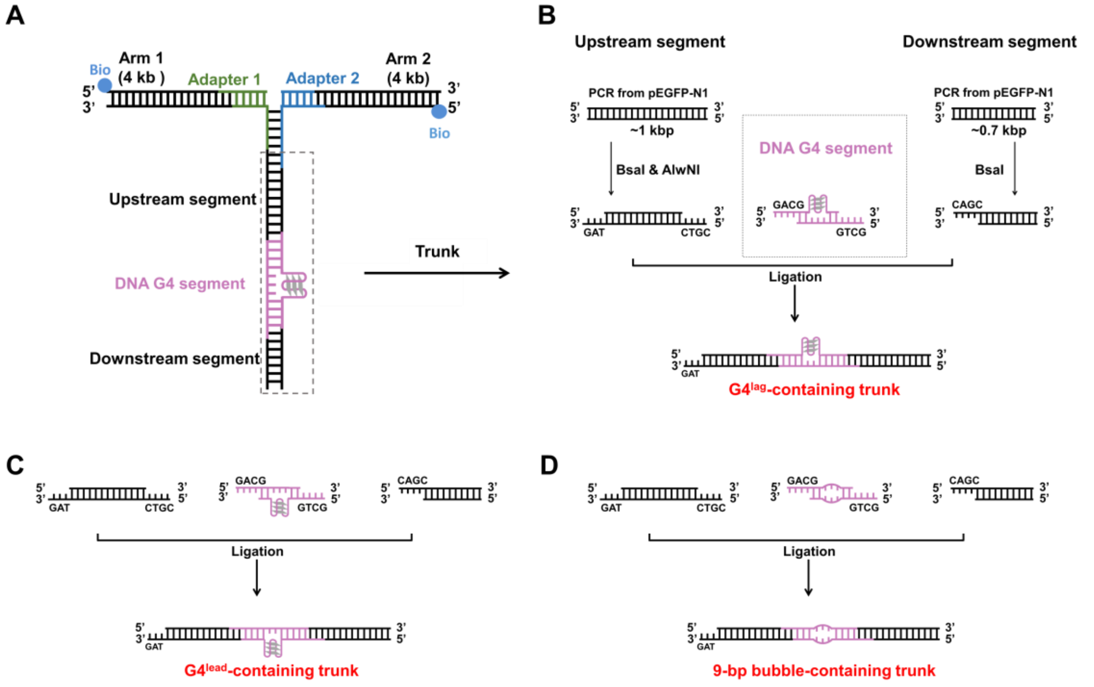

Figure S2. Construction of the T-shaped DNA template.

A. Schematic of the construction of the T-shaped DNA template. The DNA construct is composed of three DNA segments - two arms and a trunk - linked through two short adapters. Briefly, a 4-kbp arm 1 and a 4-kbp arm 2 were PCR-amplified from lambda DNA using a biotin-labeled primer. The resulting DNA fragments were digested with BstXI (NEB) to create an overhang and were subsequently ligated to a short DNA with a complementary overhang formed by adapters 1 and 2. The ligation products were annealed to create a short 35-bp trunk with a 3-bp overhang for trunk ligation. The trunk contains an embedded G4 structure (purple). The T-shaped template used for the DNA unzipping assay was constructed similarly. Briefly, a 2.2-kbp arm 3 was amplified from pBR322 using a biotin-labeled primer, while a 2.2-kbp arm 4 was amplified using a digoxigenin-labeled primer. **B.** The trunk consists of three segments (the upstream segment, the G4 segment, and the downstream segment). The 1.1-kbp upstream segment was amplified from the plasmid pEGFP-N1 and digested with AlwNI and BsaI (NEB). The $G4^{lag}$ segment was formed by annealing an oligonucleotide containing the G4 motif with an oligonucleotide

containing a 9-bp mismatched sequence. The 0.7-kbp downstream segment was amplified from the plasmid pEGFP-N1 and digested with BsaI (NEB). The final product was generated by ligating the arms with the trunk (three segments) at a 1:3:9:27 ratio. All DNA products were purified with a SanPrep Column PCR Product Purification Kit (Sangon Biotech). **C.** Diagram of DNA containing the leading strand G4. The $G4^{lead}$ segment was created similarly. **D.** Diagram of DNA containing a 9-bp bubble. In this DNA template, the G4 segment was replaced with the 9-bp bubble segment, created by annealing two oligonucleotides containing a 9-bp mismatched sequence. The DNA sequences used for constructing these DNA templates are listed in **Table S1**.

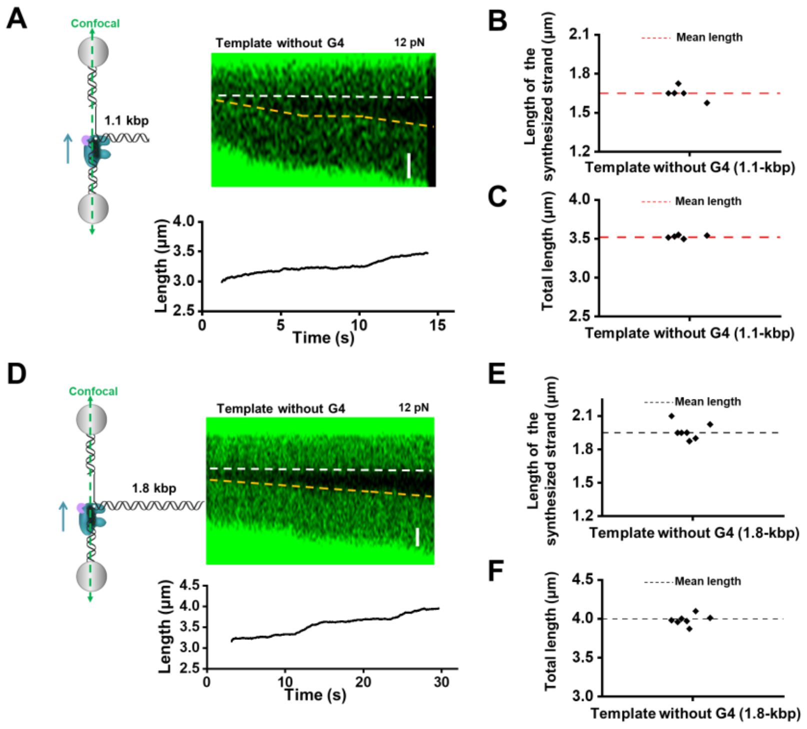

Figure S3. Determination of the total and synthesized DNA lengths when T7 DNAP stalled at G4 or overcame it.

A and D. A representative kymograph of a 1.1-kbp or 1.8-kbp intact T-shaped DNA (no G4) under 12 pN in the presence of 100 nM T7 DNAP and its corresponding DNA length. The white dotted lines indicate the boundary between ssDNA and dsDNA. The yellow dotted lines indicate the position of the replication fork. The scale bar represents 0.5 μm. **B and E.** Statistics of the synthesized DNA length after T7 DNAP completed the DNA synthesis on the 1.1-kbp or 1.8-kbp trunk. This calculation is based on the kymograph analysis. The red dotted lines represent the average length. **C and F.** Statistics of total DNA length after T7 DNAP completed the DNA synthesis on the 1.1-kbp or 1.8-kbp trunk. The black dotted lines represent the average length.

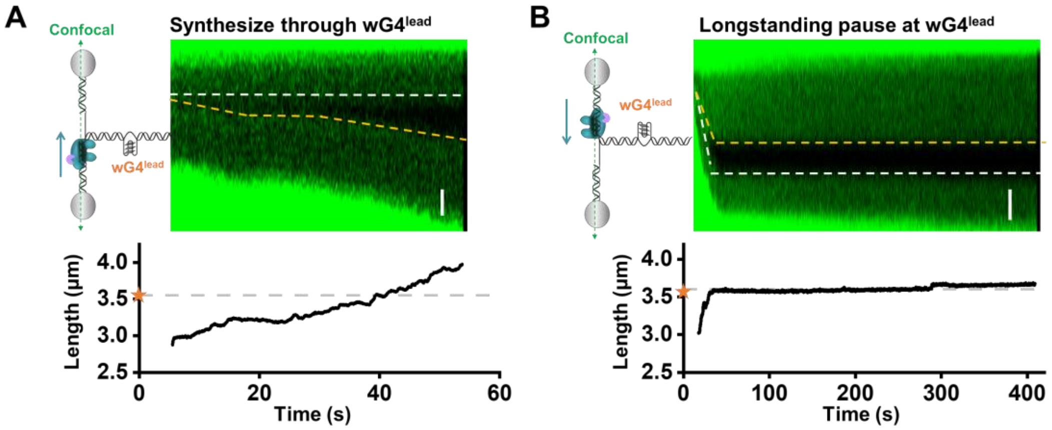

Figure S4. T7 DNAP overcomes or stalls at a wG4 in the strand displacement DNA synthesis assay.

A. A representative kymograph of a wG4^{lead}-containing DNA under 12 pN in the presence of 100 nM T7 DNAP and its corresponding DNA length showing that T7 DNAP overcomes a wG4^{lead}. **B**. A representative kymograph of a wG4^{lead}-containing DNA under 12 pN in the presence of 100 nM T7 DNAP and its corresponding DNA length showing that T7 DNAP was impeded by a wG4^{lead} in the strand displacement DNA synthesis assay after a longstanding pause. The orange pentagram indicates the wG4^{lead} location. The white dotted lines indicate the boundary between ssDNA and dsDNA. The yellow dotted lines indicate the position of the replication fork. The scale bar represents 0.5 μm.

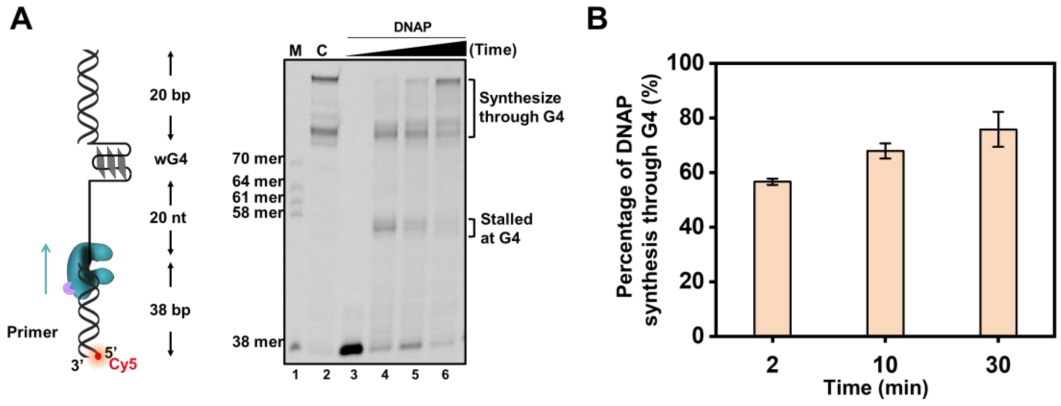

Figure S5. The primer extension of T7 DNAP along a wG4 DNA template containing dsDNA downstream of the wG4.

A. Schematic representation of the primer extension of T7 DNAP on a DNA template containing dsDNA downstream of a wG4. Denaturing PAGE analysis of the primer extension by T7 DNAP on either a control template (no G4, denoted as ‘C’) or a G4-containing DNA template. **B.** Quantitative analysis of G4-overcoming products. Data are mean ± SD from three independent experiments.

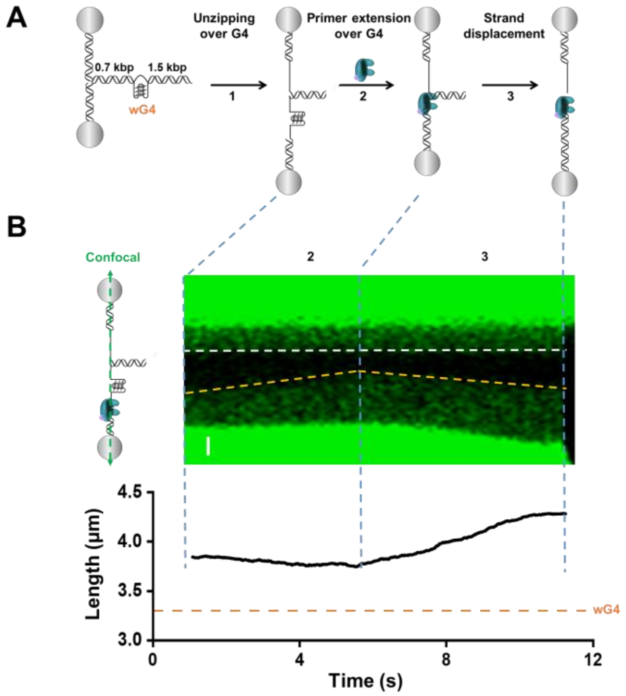

Figure S6. Single-molecule experiments reveal the G4 tolerance by T7 DNAP after the fork removal.

A. Schematic representation of the single-molecule configuration of T7 DNAP extending the primer on a wG4-containing DNA template. The experimental procedures are: (1) The upstream and G4-containing DNA segments within the trunk are mechanically unzipped to create a G4-containing ssDNA. (2) The DNA template is transferred to the T7 DNAP channel for the primer extension under 12 pN. (3) After the T7 DNAP encountered the fork, it began the strand displacement DNA synthesis under 12 pN. **B.** A representative kymograph of the DNA under 12 pN in the presence of 100 nM T7 DNAP and its corresponding DNA length. The white dotted lines indicate the boundary between ssDNA and dsDNA. The orange dotted orange line represents the position of wG4. The yellow dotted lines indicate the position of the replication fork. The scale bar represents 0.5 μm. The strand displacement DNA synthesis monitored here implied that T7 DNAP overcomes the G4 structure during the primer extension.

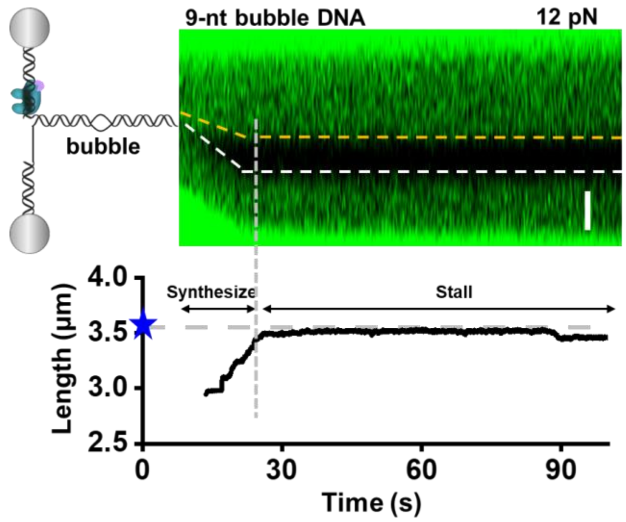

Figure S7. The strand displacement DNA synthesis of T7 DNAP on the 9-bp "bubbled" DNA template.

Schematic of the 9-bp "bubbled" template showing the 9-bp bubble position in the DNA (not to scale). A representative kymograph of a 9-bp bubble-containing DNA under 12 pN in the presence of 100 nM T7 DNAP and its corresponding DNA length are shown. The blue pentagram represents the bubble location. The white dotted lines indicate the boundary between ssDNA and dsDNA. The yellow dotted lines indicate the position of the replication fork. The scale bar represents 0.5 μm. T7 DNAP was also found to stall at the bubble position.

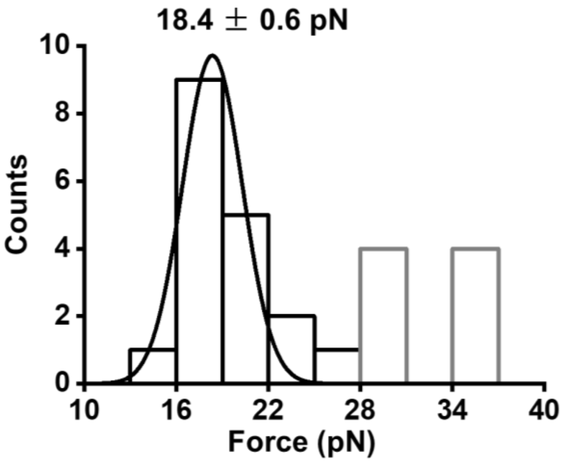

Figure S8. The disruption force of DNA around the G4 position in the presence of T7 DNAP.

The histogram of the disruption force of inactive T7 DNAP binding to DNA around the $wG4^{lag}$ position is shown. The data and the Gaussian fit are shown. Higher disruption forces (over 28 pN, grey) beyond the Gaussian fitting could result from the multiple DNAPs binding to the G4-adjacent fork junctions.

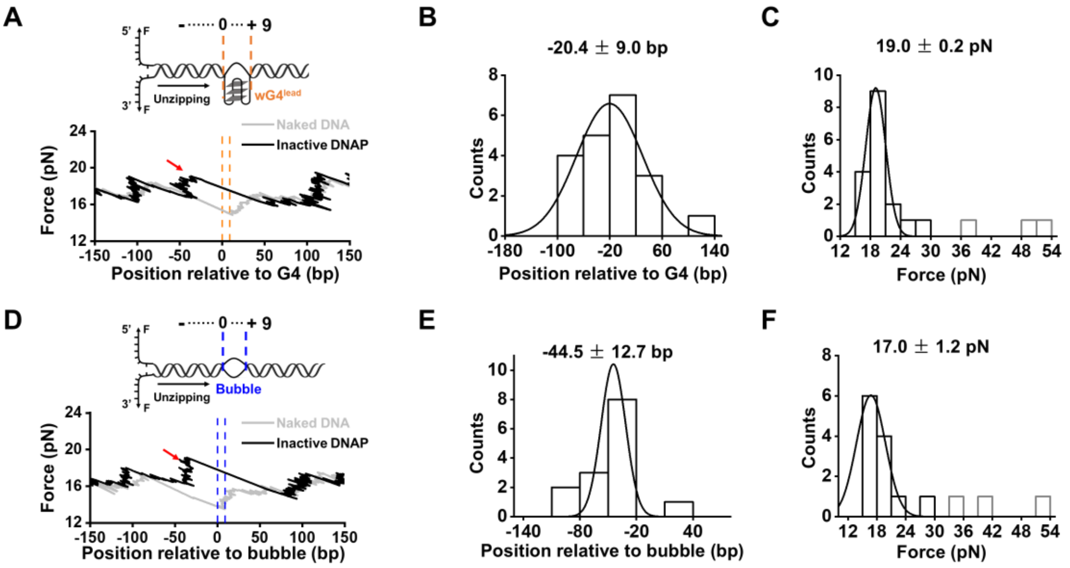

Figure S9. The DNA unzipping assays with the wG4^{lead} and the 9-bp "bubbled" DNA templates.

A. A representative DNA unzipping trace of the wG4^{lead}-containing DNA template in the presence of inactive DNAP showing the force versus number of base pairs unzipped (black). The naked DNA unzipping signatures are presented for comparison (grey). The red arrow indicates the force peak. The dotted orange lines highlight the wG4^{lead} location. **B.** The histogram of the positions of the disruption force along the wG4^{lead}-containing DNA sequence. **C.** Histogram of the disruption force around the wG4^{lead} position. **D.** A representative DNA trace of the 9-bp "bubbled" DNA in the presence of inactive DNAP showing the force versus number of base pairs unzipped (black). The naked DNA unzipping signatures are presented for comparison (grey). The red arrow indicates the force peak. The dotted blue lines represent the location of the 9-bp bubble. **E.** The histogram of the positions of the disruption force along the 9-bp bubble-containing DNA sequence. **F.** Histogram of the disruption force around the bubble position.

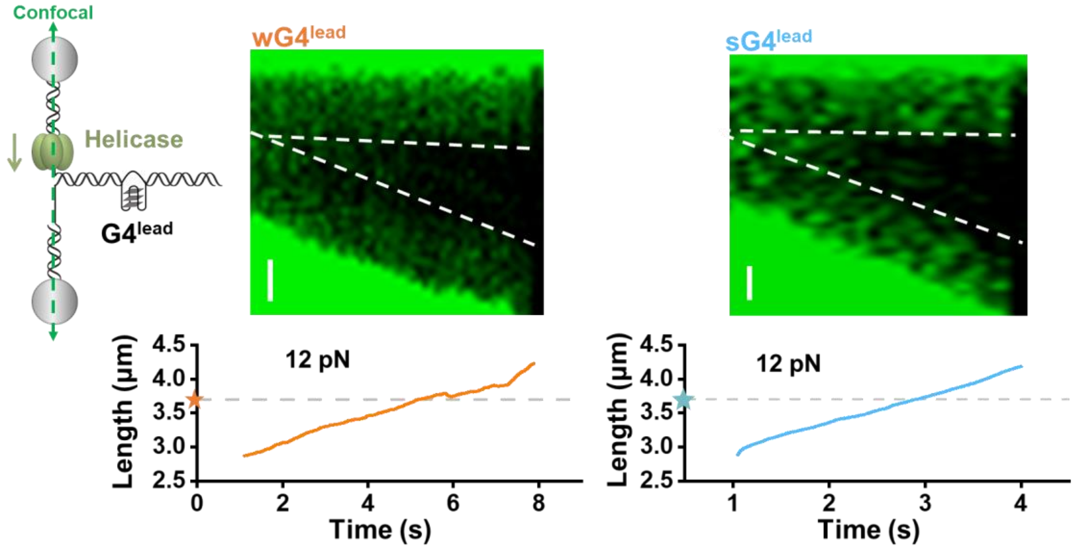

Figure S10. T7 Helicase unwinding through G4^{lead} in the optical tweezers-based assay.

A schematic of the G4^{lead} template shows the position of the G4^{lead} in the DNA (not to scale). Representative kymographs of a G4^{lead}-containing DNA under 12 pN in the presence of 10 nM T7 helicase (hexamer) and their corresponding DNA lengths show T7 helicase smoothly unwinding through G4^{lead}. The orange pentagrams represent the G4 locations. The white dotted lines highlight the boundary between ssDNA and dsDNA. The scale bar represents 0.5 μm.

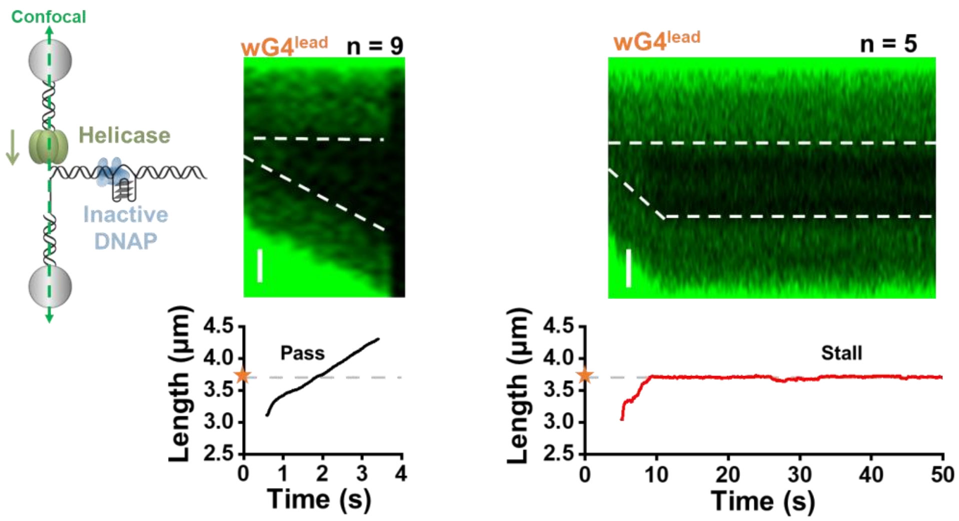

Figure S11. T7 Helicase unwinds the wG4^{lead} template in the presence of inactive T7 DNAP.

Representative kymographs of a wG4^{lead}-containing DNA under 12 pN in the presence of 10 nM T7 helicase (hexamer) and 100 nM inactive T7 DNAP and their corresponding DNA lengths are shown. The orange pentagrams represent the G4 locations. The white dotted lines indicate the boundary between ssDNA and dsDNA. The scale bar represents 0.5 μm.

These results suggest that T7 helicase has a chance to dismantle the inactive T7 DNAPs at the wG^{lead}-induced DNA fork and continually unwind downstream dsDNA.

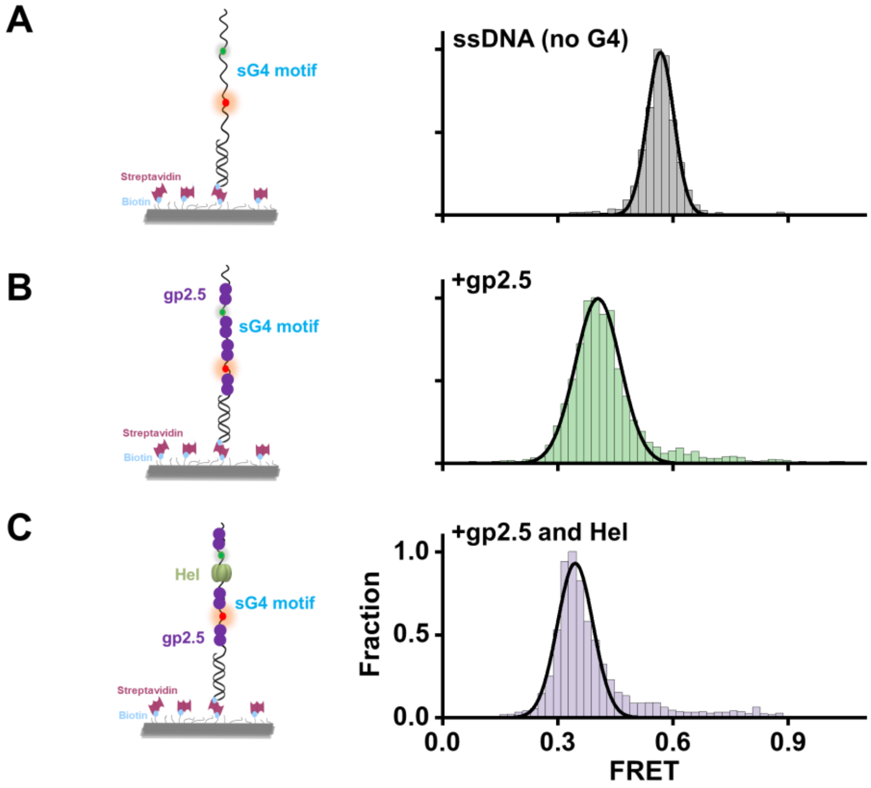

Figure S12. The FRET histograms before and after gp2.5 and helicase binding to ssDNA contain no G4 structure.

A. The FRET distribution shows the sG4 motif in the Na^{+}-depleted buffer. The G4 structure is not formed in this condition, and the *E* value is centered at ~ 0.56. **B.** The FRET distribution shows the DNA after the addition of 2 μM gp2.5. In this condition, the *E* value is centered at ~ 0.40. **C.** The FRET distribution shows the DNA after adding 2 μM gp2.5 and 30 nM helicase hexamer. The binding of the two proteins to the ssDNA makes the ssDNA less flexible, thus decreasing the *E*. This FRET state corresponds to the unwound G4 bound by the two proteins.

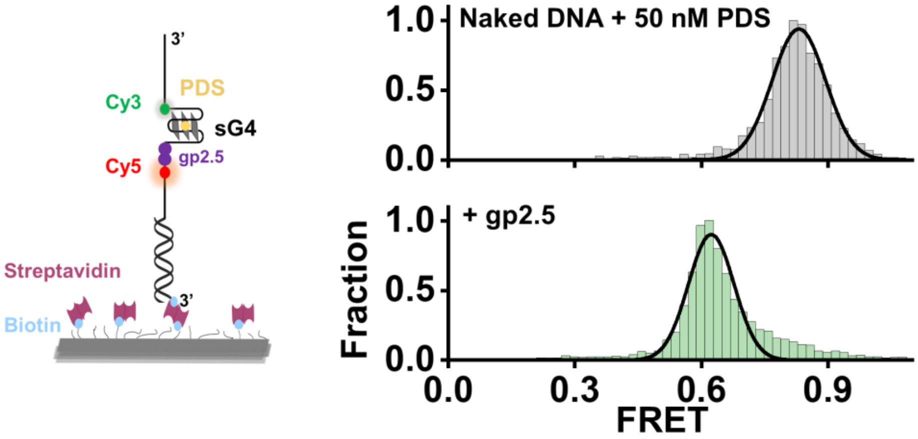

Figure S13. The FRET histograms of the DNA substrate in the presence of gp2.5 and 50 nM PDS.

Schematic diagram of the fluorescently labeled DNA harboring a sG4 motif. The upper panel shows the FRET distribution of the DNA in the presence of 50 nM PDS. The lower panel shows the FRET distribution of the DNA substrate in the presence of 50 nM PDS and 2 μM gp2.5. The company of PDS ensures the formation of the G4 structure and prevents the G4 destabilization by gp2.5. Therefore, the detected decrease in *E* is attributed to the gp2.5 binding to the G4-adjacent ssDNA instead of G4 unwinding.

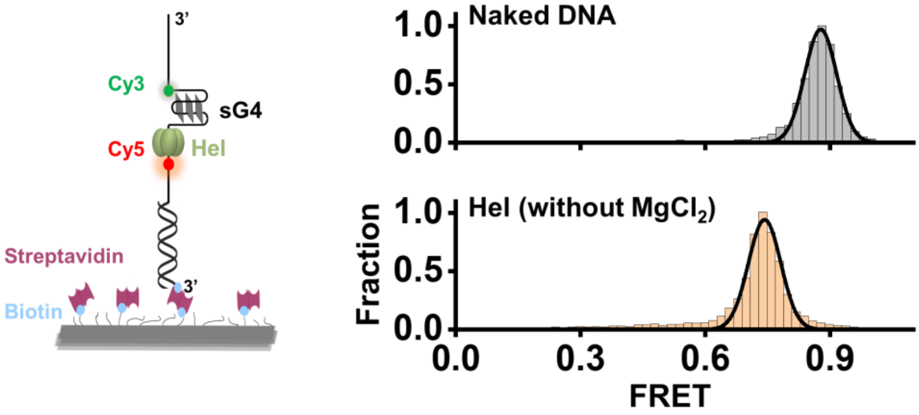

Figure S14. The FRET histogram of the G4-containing DNA substrate after T7 helicase binds to G4-adjacent ssDNA without unwinding.

A schematic diagram of T7 helicase binding to the fluorescently labeled DNA harboring the sG4 motif without unwinding is shown on the left. The upper panel shows the FRET distribution of the sG4-containing substrate in the T7 reaction buffer without $MgCl_2$. The lower panel shows the FRET distribution of the sG4-containing substrate in the presence of 30 nM helicase (hexamer) in the absence of $MgCl_2$. The data and their Gaussian fits are shown. The helicase unwinding activity is abolished due to the lack of Mg^{2+}. The FRET state of ~ 0.75 represents the binding of T7 helicase to the substrate.

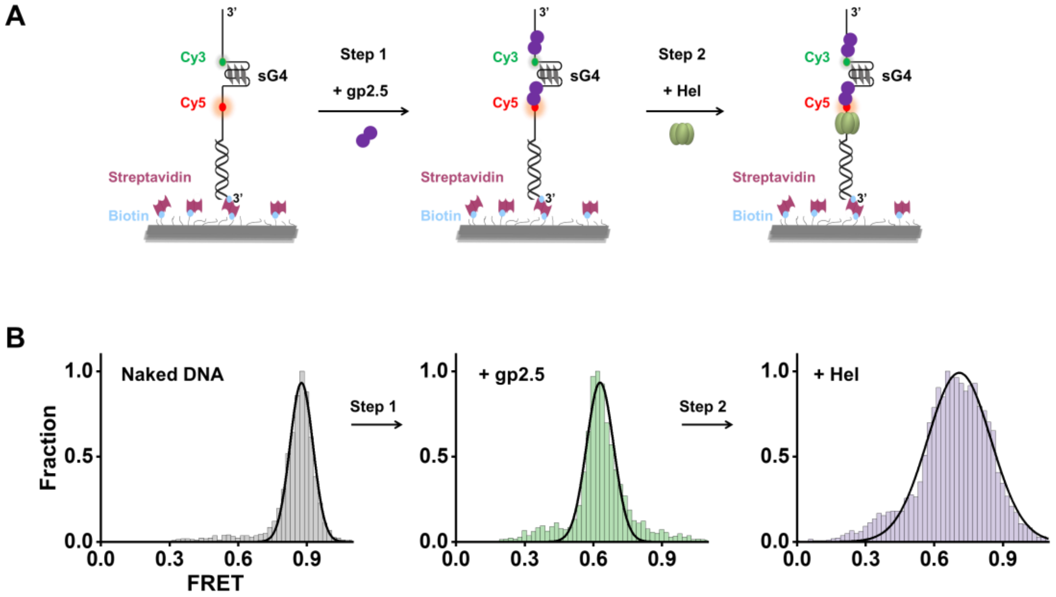

Figure S15. The FRET histograms of the DNA before and after the sequential introduction of gp2.5 and T7 helicase.

A. A schematic diagram of the experimental procedure. The sG4-containing DNA substrate was introduced by gp2.5 first, followed by the introduction of T7 helicase (Hel). **B.** The corresponding FRET distributions and the Gaussian fittings (black) after the sequential introduction of gp2.5 and T7 helicase. Gp2.5 binds to the G4-adjacent ssDNA, resulting in a decrease in the *E* from 0.87 to 0.64. Following that, introducing T7 helicase broadened the FRET distribution and caused a slight shift to the right, whereas the low *E* values did not show up. These results indicate that the G4 structure is not unwound in this condition due to the prevention of helicase loading by the pre-binding of gp2.5 to the G4-adjacent ssDNA.

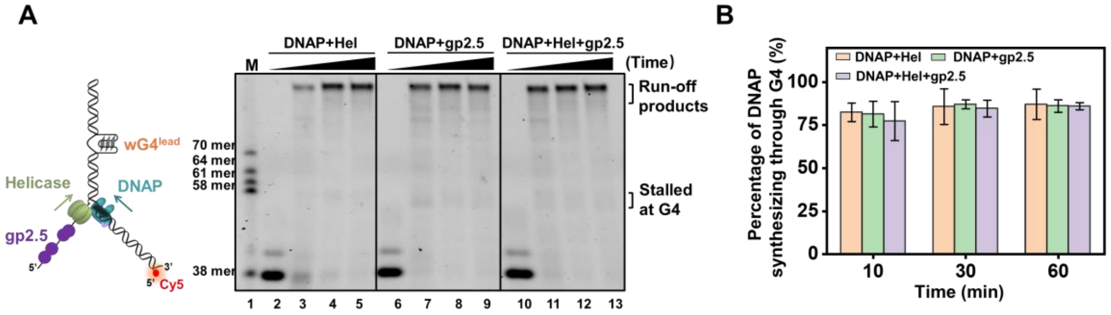

Figure S16. T7 DNAP replicates wG4^{lead}-containing DNA in the presence of T7 helicase and/or gp2.5.

A. Schematic illustrates the ensemble leading-strand DNA replication assay with a wG4^{lead}-containing template in the presence of T7 helicase and/or gp2.5. A representative gel under indicated experimental conditions is shown. **B.** The percentage of G4-overcoming replication products in 10, 30, and 60 min. Data are mean ± SD from three independent experiments.

Table S1. Sequences of the oligonucleotides and primers for the construction of the DNA templates.

Figures	Names	Sequence
Figure S1A	wG4-ssDNA	5’-CGGTATTTCACACCG-biotin-3’<br>5’-CGGTGTGAAATACCGACAC/iCy5dT/GGGTTAGGGTTAGGGTTAGGG/iCy3/TCAGCCAGCAAGACGTAGCT-3’
Figures 5 & S1B	sG4-ssDNA	5’-CGGTATTTCACACCG-biotin-3’<br>5’-CGGTGTGAAATACCGACACTCCAGGAGCT/iCy5/CACCATGGGTGGGTGGGTGGG/iCy3/TCAGCCAGCAAGACGTAGCT-3’
Figure S1C	wG4-partially match	5’-AGCTACGTCTTGCTGGCTGAATAGTTAGAAGTGTCGGTATTTCACACCG-biotin-3’<br>5’-CGGTGTGAAATACCGACAC/iCy5dT/GGGTTAGGGTTAGGGTTAGGG/iCy3/TCAGCCAGCAAGACGTAGCT-3’
Figure S1D	sG4-partially match	5’-AGCTACGTCTTGCTGGCTGAATAGTTAGAATGGTGAGCTCCTGGAGTGTCGGTATTTCACACCG-biotin-3’<br>5’-CGGTGTGAAATACCGACACTCCAGGAGCT/iCy5/CACCATGGGTGGGTGGGTGGG/iCy3/TCAGCCAGCAAGACGTAGCT-3’
Figure S1E	wG4-fully match	5’-AGCTACGTCTTGCTGGCTGACCCTAACCCTAACCCTAACCCAGTGTCGGTATTTCACACCG-biotin-3’<br>5’-CGGTGTGAAATACCGACAC/iCy5dT/GGGTTAGGGTTAGGGTTAGGG/iCy3/TCAGCCAGCAAGACGTAGCT-3’
Figures 1, 2, 3, S3, S4, S6, S7, S10 & S11	Arm 1	5’-biotin-TGATAAGCAGAATGGCATCGTTCC-3’<br>5’-CTGCAGAACCATTGACATGGATCACTGTTGATTCTCGCTGTCA-3’
	Arm 2	5’-biotin-TGATAAGCAGAATGGCATCGTTCC-3’<br>5’-CTGCAGAACCATACTGATGGATCACTGTTGATTCTCGCTGTCA-3’
Figures 2 & S7	Arm 3-unzipping	5’-biotin-CGAAATAGACAGATCGCTGAG-3’<br>5’-ATTACCATTGACATGGCAACTTTATCCGCCTCCA-3’
	Arm 4-unzipping	5’-dig-CGAAATAGACAGATCGCTGAG-3’<br>5’-ATTACCACGAATTTGGCATCCGCTTACAG-3’
Figures 1, 2, 3, S3, S4, S6, S7, S10 & S11	Adapter 1	5’/phos/CGATGCAGTACCGAGCTCATCCAATTCTACATGCCGC-3’<br>5’/phos/GCCTTGCACGTGATTACGAGATATCGATGATTGCGGCGGCATGTAGAATTGGATGAGCTCGGTACTGCATCGTGAC-3’
	Adapter 2	5’-CGTTACGTCATTCTATACACTGTACAGCTTACACTG-3’<br>5’/phos/GTAACCTGTACAGTGTATAGAATGACGTAACGCGCAATCATCGATATCTCGTAATCACGTGCAAGGCCTA-3’
	Upstream segment	5’-TTAACAGCTACTGGGTCATTAGTTCATAGCCCA-3’<br>5’-ACTGGTCTCACGTCCTCCTTGAAGTCGATGCCCTTCA-3’

	Downstream segment	5'-ACTGGTCTCACAGCCACAACGTCTATATCATGGCCGA-3'<br>5'-CACTCAACCCTATCTCGGTCTATTCTT-3'
	OT-wG4^{lead}	5'/phos/GCTGCGGTGTGAAATACCGACACAGGGTTAGGGTTAGGGTTAGGGACAGCCAGCAAGACGTAGCT-3'<br>5'/phos/GACGAGCTACGTCTTGCTGGCTGTATAGTTAGATGTGTCGGTATTTCACACCG-3'
	OT-wG4^{lag}	5'/phos/GACGCGGTGTGAAATACCGACACAGGGTTAGGGTTAGGGTTAGGGACAGCCAGCAAGACGTAGCT-3'<br>5'/phos/GCTGAGCTACGTCTTGCTGGCTGTATAGTTAGATGTGTCGGTATTTCACACCG/-3'
	OT-sG4^{lead}	5'/phos/GCTGCGGTGTGAAATACCGACACAGGGTGGGTGGGTGGGACAGCCAGCAAGACGTAGCT-3'<br>5'/phos/GACGAGCTACGTCTTGCTGGCTGTATAGTTAGATGTGTCGGTATTTCACACCG-3'
	OT-sG4^{lag}	5'/phos/GACGCGGTGTGAAATACCGACACAGGGTTAGGGTTAGGGTTAGGGACAGCCAGCAAGACGTAGCT-3'<br>5'/phos/GCTGAGCTACGTCTTGCTGGCTGTATAGTTAGATGTGTCGGTATTTCACACCG-3'
Figures S7 & S9	9-bp bubble	5'/phos/GACGCGGTGTGAAATACCGACACAATAGTTAGAACAGCCAGCAAGACGTAGCT-3'<br>5'/phos/GCTGAGCTACGTCTTGCTGGCTGTATAGTTAGATGTGTCGGTATTTCACACCG-3'
Figures 1B & 6C	Gel-PE-wG4	5'/Cy5/CGAAGGCTACGTCCAGGAGCGCACCATCTTCTTCAAGG-3'<br>5'-CGGTGTGAAATACCGACACAGGGTTAGGGTTAGGGTTAGGGACAGCCAGCAAGACGTAGCTCCTTGAAGAAGATGGTGCGCTCCTGGACGTAGCCTTCG-3'
	Gel-PE-sG4	5'/Cy5/CGAAGGCTACGTCCAGGAGCGCACCATCTTCTTCAAGG-3'<br>5'-CGGTGTGAAATACCGACACAGGGTGGGTGGGTGGGACAGCCAGCAAGACGTAGCTCCTTGAAGAAGATGGTGCGCTCCTGGACGTAGCCTTCG-3'
Figure S5	Gel-wG4	5'/Cy5/CGAAGGCTACGTCCAGGAGCGCACCATCTTCTTCAAGG-3'<br>5'-CGGTGTGAAATACCGACACAGGGTTAGGGTTAGGGTTAGGGACAGCCAGCAAGACGTAGCTCCTTGAAGAAGATGGTGCGCTCCTGGACGTAGCCTTCG-3'<br>5'-TGTGTCGGTATTTCACACCG-3'
Figure 4	Gel-wG4^{lead}	5'/Cy5/CGAAGGCTACGTCCAGGAGCGCACCATCTTCTTCAAGG-3'<br>5'-CGGTGTGAAATACCGACACAGGGTTAGGGTTAGGGTTAGGGACAGCCAGCAAGACGTAGCT CCTTGAAGAAGATGGTGCGCTCCTGGACGTAGCCTTCG-3'<br>5'-AACGCCAAGCCAGGTATAAAGCATGGAGGGACACGGCGAGCTACGTCTTGCTGGCTGTATAGTTAGATGTGTCGGTATTTCACACCG-3'
	Gel-wG4^{lag}	5'/Cy5/CGAAGGCTACGTCCAGGAGCGCACCATCTTCTTCAAGG-3'<br>5'-CGGTGTGAAATACCGACACAATAGTTAGAACAGCCAGCAAGACGTAGCTCCTTGAAGAAGATGGTGCGCTCCTGGACGTAGCCTTCG-3'<br>5'-AACGCCAAGCCAGGTATAAAGCATGGAGGGACACGGCGAGCTACGTCTTGCTGGCTGTGGGTTAGGGTTAGGGTTAGGGTGTGTCGGTATTTCACACCG-3'

	Gel-sG4^{lag}	5'/Cy5/CGAAGGCTACGTCCAGGAGCGCACCATCTTCTTCAAGG-3'<br>5'-CGGTGTGAAATACCGACACAATAGTTAGAACAGCCAGCAAGACGTAGCTCCTTGAAGAAGATGGTGCGCTCCTGGACGTAGCCTTCG-3'<br>5'- AACGCCAAGCCAGGTATAAAGCATGGAGGGACACGGCGAGCTACGTCTTGCTGGCTGTGGGTGGGTGGGTGGGTGTGTCGGTATTTCACACCG-3'
Figures 4 & 6	Gel-sG4^{lead}	5'/Cy5/CGAAGGCTACGTCCAGGAGCGCACCATCTTCTTCAAGG-3'<br>5'-CGGTGTGAAATACCGACACAGGGTGGGTGGGTGGGACAGCCAGCAAGACGTAGCT CCTTGAAGAAGATGGTGCGCTCCTGGACGTAGCCTTCG-3'<br>5'-AACGCCAAGCCAGGTATAAAGCATGGAGGGACACGGCGAGCTACGTCTTGCTGGCTGTATAGTTAGATGTGTCGGTATTTCACACCG-3'
Figure 3	Gel-wG4-unwinding	5'-CGGTGTGAAATACCGACACAATAGTTAGAACAGCCAGCAAGACGTAGCTCCTTGAAGAAGATGGTGCGCTCCTGGACGTAGCCTTCG/Cy5/-3'<br>5'-AACGCCAAGCCAGGTATAAAGCATGGAGGGACACGGCGAGCTACGTCTTGCTGGCTGTGGGTTAGGGTTAGGGTTAGGGTGTGTCGGTATTTCACACCG-3'
	Gel-sG4-unwinding	5'-CGGTGTGAAATACCGACACAATAGTTAGAACAGCCAGCAAGACGTAGCTCCTTGAAGAAGATGGTGCGCTCCTGGACGTAGCCTTCG/Cy5/-3'<br>5'-AACGCCAAGCCAGGTATAAAGCATGGAGGGACACGGCGAGCTACGTCTTGCTGGCTGTGGGTGGGTGGGTGGGTGTGTCGGTATTTCACACCG-3'

G4 motifs are highlighted with underlines. PE and OT represent primer extension and optical tweezers, respectively.
